# T cell–nanodrug conjugates synchronize vascular normalization and immune activation for solid tumor therapy

**DOI:** 10.1126/sciadv.adp2955

**Published:** 2026-08-07

**Authors:** Xin Yang, Ye Su, Xiaoyun Ye, Changjiang Liu, Xin Zhao, Yuyan Su, Qingyu Dong, Wenxuan Zeng, Xinghua Sui, Xiuman Zhou, Guanyu Chen, Yujia Zhang, Dalin Wu, Juan Liu

**Affiliations:** ^1^ School of Pharmaceutical Sciences (Shenzhen), Shenzhen Campus of Sun Yat-sen University, Shenzhen 518107, China.; ^2^ School of Biomedical Engineering, Shenzhen Campus of Sun Yat-sen University, Shenzhen 518107, China.; ^3^ Laboratory for Bio-Iontronics (BION), Institute of Electrical and Micro Engineering, School of Engineering, École Polytechnique Fédérale de Lausanne (EPFL), Lausanne 1015, Switzerland.

## Abstract

Adoptive T cell therapy requires T cells to infiltrate vascular tissues and preserve immune function. In solid tumor treatment, however, the surrounding microenvironment produces abnormal vasculature that impedes T cell infiltration. An approach that enables vascular normalization and enhances adoptive T cell function in parallel is essential for effective therapy but has not been reported. Here, we report the use of lenvatinib (LEN) to induce transient vascular normalization, thereby facilitating T cell infiltration. Moreover, LEN enhances T cell persistence by promoting the differentiation of T cells toward a memory phenotype. Our results indicate that the differentiation is by suppressing the PI3K–AKT–mTOR pathway, which drives effector differentiation, and by activating FOXO1, a transcription factor that promotes memory formation. To coordinate the transient vascular normalization and T cell enhancement, we link LEN-loaded, PD-L1–blocking micelles to T cells through acid-labile click chemistry, forming pH-responsive T cell–nanodrug conjugates. The conjugates synchronize the intratumoral release of LEN and the PD-L1 antagonist peptide OPBP-1, thereby coordinating vascular normalization, T cell differentiation, and checkpoint blockade. In vivo, the conjugates increased intratumoral CD8^+^ T cells and splenic memory T cells by over sixfold in B16-OVA tumors and achieved complete regression in a subset of MC38-OVA tumors without systemic toxicity, providing a promising strategy for solid tumor immunotherapy.

## Teaser

T cell–nanodrug conjugates coordinate vascular normalization and T cell memory differentiation to enhance antitumor immunity.

## INTRODUCTION

Adoptive T cell therapy (ACT) is a type of immunotherapy that uses a patient’s own T cells to treat diseases. However, current ACT approaches are ineffective against solid tumors (*1*, *2*) due to the structural barriers posed by immunosuppressive tumor microenvironments (*3*–*5*) and abnormal vasculature (*6*). Endothelial cells down-regulate adhesion molecules, such as intercellular adhesion molecules (ICAM-1) and vascular cell adhesion molecules (VCAM-1), and release inhibitory angiocrines, including interleukin-6 (IL-6), prostaglandin E_2_ (PGE_2_), and IL-10, forming an “immune-excluded vascular niche” (*7*–*10*). Low-dose vascular endothelial growth factor receptor (VEGFR) blockade can induce localized and short-lived improvements in vessel structure and oxygenation, briefly enhancing vascular perfusion and immune accessibility. However, the normalization effect is temporary and difficult to synchronize with the kinetics of T cell activation and trafficking (*11*–*14*), demanding a spatiotemporal coordination between vascular and immune modulation.

The differentiation states of T cells determine the therapeutic persistence and antitumor efficacy (*15*). Central memory T cells (TCMs) exhibit superior proliferative potential, metabolic fitness, and longevity by comparison with more differentiated subsets, such as terminal effector T cells (*16*, *17*). Therefore, TCMs play a pivotal role in sustaining long-term antitumor immunity and in ACT models (*18*, *19*). Approaches that can promote T cell differentiation into TCM become critical for extending the therapeutic durability of ACT (*20*).

Lenvatinib (LEN) is a multitargeted tyrosine kinase inhibitor (TKI) of VEGFR (*21*), fibroblast growth factor receptors (FGFRs) (*22*), and platelet-derived growth factor receptors (PDGFRs) (*23*). LEN has shown synergistic effects with programmed cell death protein 1 (PD-1) blockade in clinical studies, suggesting immunomodulatory activity beyond vascular remodeling (*24*–*26*). Here, in addition to promoting vascular normalization in tumor tissues, we identified that LEN can also induce TCM formation, which correlates with suppression of the phosphatidylinositol 3-kinase (PI3K)–protein kinase B (AKT)–mechanistic target of rapamycin (mTOR) pathway and reduced phosphorylation of forkhead box protein O1 (FOXO1). On the basis of the discovery, we developed a tumor-responsive T cell–nanodrug conjugate that coordinates the dual vascular–immune modulatory effect of LEN to overcome the T cell infiltration and function demands of ACT. Our self-coordinated conjugates provide a spatiotemporally coupled ACT approach that enhances T cell infiltration, persistence, and antitumor efficacy for solid tumor immunotherapy.

## RESULTS

### Fabrication and characterization of T cell–LEN-nOPBP-1 (T-LEN-nOPBP-1) conjugates

**Fig. 1.**
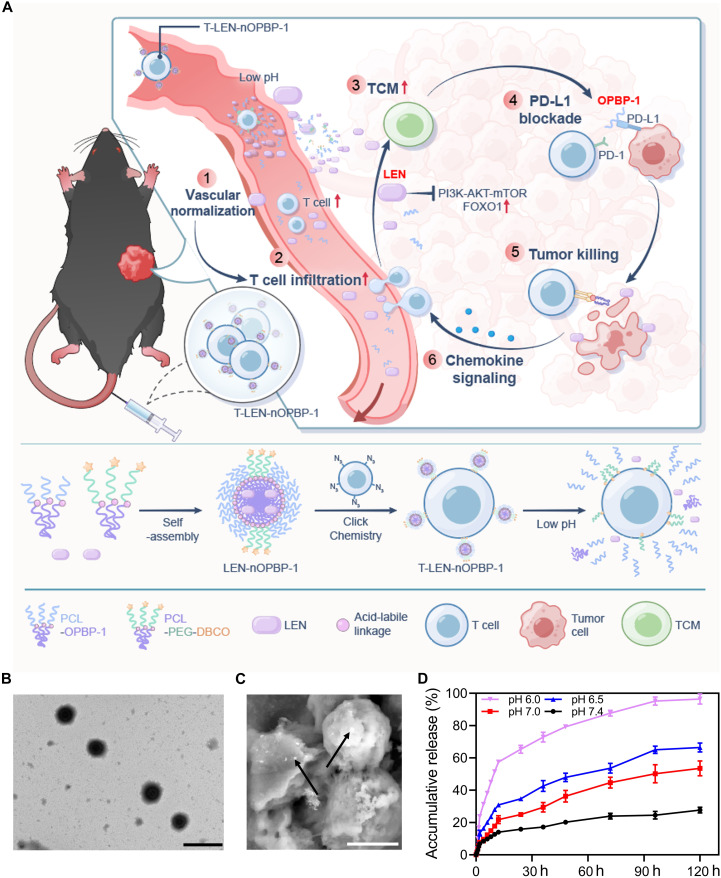
Construction and coordinated mechanism of the T cell–nanodrug conjugates. (**A**) Acid-responsive LEN-nOPBP-1 micelles are linked to T cells through click chemistry (see Materials and Methods). At the tumor location, the acidic microenvironment triggers the release of LEN and OPBP-1, initiating six coordinated processes: (i) vascular normalization that restores perfusion and endothelial adhesiveness, (ii) increased T cell infiltration, (iii) T cell differentiation toward the TCM phenotype through PI3K–AKT–mTOR inhibition and FOXO1 activation by LEN, (iv) PD-L1 blockade by OPBP-1 peptides, (v) tumor-cell killing by activated T cells, and (vi) reinforcement of a pro-infiltrative chemokine-adhesion feedback loop among T cells, endothelium, and tumor cells. (**B**) Transmission electron microscopy image showing the spherical morphology of LEN-nOPBP-1 micelles. Scale bar, 200 nm. (**C**) Scanning electron microscopy image revealing micelles attached to the surface of T cells. Scale bar, 5 μm. This image corresponds to a higher-magnification view of the representative field shown in fig. S9. (**D**) In vitro release profiles of LEN–nOPBP-1 micelles under different pH conditions (pH 6.0, 6.5, 7.0, and 7.4), determined by high-performance liquid chromatography. The released LEN amount is normalized relative to the total LEN loading amount. Data are mean values ± SD; *n* = 3. h, hours.

We constructed a tumor-responsive T cell–nanodrug conjugate, which consists of acid-responsive micelles (LEN-nOPBP-1). The LEN-loaded micelles were formed by the self-assembly of poly(ε-caprolactone)–OPBP-1 (PCL–OPBP-1) and PCL–poly(ethylene glycol)–dibenzocyclooctyne (PCL–PEG–DBCO), and were covalently attached to azide-labeled T cells by strain-promoted azide-alkyne cycloaddition click chemistry (*27*–*31*). In this structure, T cells play the roles of targeted delivery carriers and therapeutic effector cells at the same time. The coupled nanodrug LEN-nOPBP-1 functions as a synergistic immunomodulatory system, in which LEN drives tumor vascular normalization and supports TCM differentiation, while OPBP-1, a PD-L1–blocking peptide, counteracts immune suppression through checkpoint blockade (*32*). Upon exposure to the acidic tumor microenvironment, the micelles disassemble and release LEN and OPBP-1, thereby enabling localized vascular normalization and sustained T cell activation (Fig. 1A).

The LEN-nOPBP-1 micelles were self-assembled from the two three-armed PCL-based amphiphiles. The configuration of two amphiphiles is necessary to decouple drug release from surface anchoring. The PCL–OPBP-1 allows OPBP-1 peptide release for checkpoint blockade while preventing OPBP-1 immobilization on the T cell membrane. The PCL–PEG–DBCO introduces a DBCO moiety for bioorthogonal conjugation to azide–labeled T cells. The long PEG chains in PCL–PEG–DBCO ensure adequate DBCO exposure for efficient conjugation (*33*–*36*). Synthetic procedures are detailed in Supplementary Notes (fig. S1), and polymer characterizations are shown in figs. S2 to S4. An optimized 16:1 ratio of PCL–OPBP-1 to PCL–PEG–DBCO was used to form micelles in this work, showing 46% PD-L1–blocking activity and efficient surface anchoring to T cells (fig. S5).

Transmission electron microscopy showed that the LEN-nOPBP-1 micelles have a spherical morphology (Fig. 1B), and dynamic light scattering showed an average hydrodynamic diameter of 171 ± 26 nm after loading LEN (fig. S6). Flow cytometry (fig. S7) and confocal microscopy (fig. S8) confirmed that the LEN-nOPBP-1 micelles anchored onto the T cell surface after 1 hour of coincubation, showing efficient micelle-cell conjugation and colocalization. Scanning electron microscopy (Fig. 1C and fig. S9) further visualized micelles distributed on the T cell membrane, indicating stable T-LEN-nOPBP-1 conjugate formation. Further, we assessed the LEN content in the supernatant of the nanoparticle preparation solution by high-performance liquid chromatography and calculated the drug loading and encapsulation efficiency based on the total LEN and micelle loading amounts. The micelles achieved 4% drug loading and 72% encapsulation efficiency. Drug-release profiling under varying pH conditions showed an acid-responsive behavior. LEN (15.8%) was released at pH 7.4 after 24 hours, whereas 65.4% was released at pH 6.0 (Fig. 1D), an acidic value typical of tumor microenvironments (*37*–*39*).

The structural design and stimuli-responsive behavior of the T cell–nanodrug conjugate directly address the intrinsic limitations associated with free drug administration. Free LEN or PD-L1–blocking peptides are typically constrained by rapid systemic clearance, insufficient tumor accumulation, and off-target exposure in vivo, which collectively limit their therapeutic efficacy. In contrast, anchoring drug-loaded micelles to the T cell surface enables high, stable drug loading while preserving drug association with T cells during circulation, thereby synchronizing drug delivery with T cell trafficking. The pH-responsive micellar structure ensures the drug stability under physiological conditions while enabling coordinated micelle disassembly and simultaneous release of LEN and OPBP-1 upon exposure to the acidic tumor microenvironment. The tumor-triggered release strategy spatially confines vascular normalization and immune checkpoint blockade to the tumor site, thereby promoting localized vascular remodeling and sustained activation of tumor-infiltrating T cells while minimizing systemic toxicity.

### T-LEN-nOPBP-1 enables vascular normalization and T cell infiltration

**Fig. 2.**
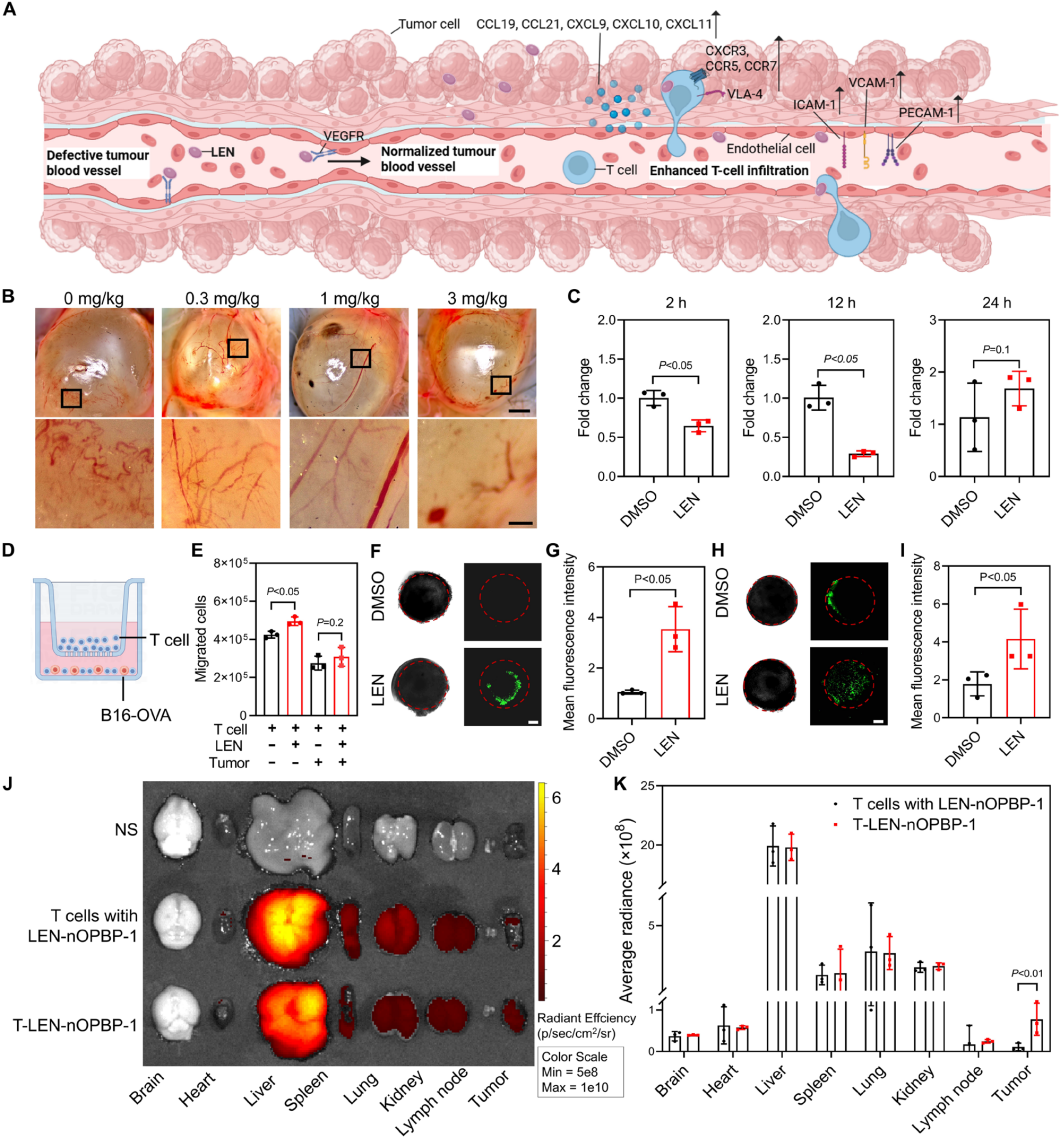
T-LEN-nOPBP-1 conjugates enable vascular normalization and in situ T cell infiltration. (**A**) LEN enhances T cell infiltration by promoting tumor vascular normalization and activating a pro-infiltrative chemokine–adhesion circuit among T cells, endothelial cells, and tumor cells. In T cells, LEN up-regulated *Vla4*, *Ccr7*, *Cxcr3*, and *Ccr5*, reinforcing migratory competence (*45*, *46*). In endothelial cells, LEN increased *Icam1*, *Vcam1*, and *Cd31*, enhancing adhesion and transmigration (*47*, *48*). Tumor cells responded by elevating *Ccl19*, *Ccl21*, *Cxcl9*, *Cxcl10*, and *Cxcl11*, generating chemokine gradients that attracted lymphocytes deep into the parenchyma (*49*–*51*). (**B**) Images of alginate-encapsulated B16-OVA tumor pellets after peri-tumoral LEN treatment in C57BL/6 mice, displaying dose-dependent vascular remodeling. Scale bars, 1 mm (upper) and 200 μm (lower). (**C**) qRT-PCR analysis of *Vegfr* expression in human endothelial cells (HUVECs) treated with 3 μM LEN, revealing transient down-regulation at 12 hours and recovery by 24 hours. (**D**) In the Transwell migration assay, activated CD8^+^ T cells were placed in the upper chamber, and LEN with or without B16-OVA cells were in the lower chamber. (**E**) Quantification of migrated CD8^+^ T cells by flow cytometry. (**F**) Confocal fluorescence imaging and (**G**) quantification of LEN-treated, carboxyfluorescein succinimidyl ester (CFSE)–labeled CD8^+^ T cells infiltrating 3D tumor spheroids at 24 hours. Scale bar, 100 μm. (**H**) Confocal fluorescence imaging and (**I**) quantification of DBCO-Cy5 labeled T cells infiltrating into LEN-conditioned tumor spheroids at 24 hours. Scale bar, 100 μm. (**J**) In vivo biodistribution of transferred T cells in B16-OVA tumor–bearing mice 12 hours after intravenous administration. Mice received normal saline (NS), free T cells, free LEN-nOPBP-1 coadministered with T cells, or T-LEN-nOPBP-1 conjugates. (**K**) Quantification of Cy5-labeled T cell distribution corresponding to (J). Data in (C), (E), (G), (I), and (K) are mean values ± SD; *n* = 3.

We hypothesized that coordinated inhibition of multiple upstream receptor tyrosine kinases (RTKs) regulating angiogenic and metabolic signaling would promote tumor vascular normalization and T cell infiltration while favoring memory-associated T cell differentiation through attenuation of RTK-driven PI3K–AKT–mTOR signaling (*40*, *41*). On the basis of this rationale, we selected LEN, a multitarget RTK inhibitor, to test whether such integrated vascular and immune modulation could be achieved in the context of adoptive cell therapy. We first examined the kinetics of LEN-induced vascular remodeling to define its relationship with T cell access to tumors (Fig. 2A).

LEN reorganized disordered endothelial junctions into structured networks in a dose- and time-dependent manner, reaching maximal normalization at 3 μM after 12 hours (fig. S10). Beyond this threshold, excess dosing or prolonged exposure destabilized the vasculature and compromised network integrity. In vivo, a similar trend emerged when LEN (1 mg kg^−1^) restored vessel alignment and perfusion, LEN (0.3 mg kg^−1^) was ineffective, and LEN (3 mg kg^−1^) caused vessel pruning and perfusion loss (Fig. 2B). Consistent with these observations, *Vegfr* transcription transiently declined at 12 hours and rebounded by 24 hours (Fig. 2C), pinpointing a short-lived normalization phase defined by the reversible VEGFR suppression effect of LEN. To determine whether these time-dependent endothelial phenotypes were associated with VEGFR signaling at the protein level, we analyzed total and phosphorylated VEGFR2 by Western blot. VEGFR2 is the principal signaling receptor mediating VEGF-driven endothelial responses (*42*, *43*). Total VEGFR2 protein levels were reduced at 2 and 12 hours and partially recovered at 24 hours, whereas VEGFR2 phosphorylation remained suppressed throughout the time course, with the strongest inhibition observed at 24 hours (fig. S11). These protein-level dynamics are consistent with the dose- and time-sensitive effects of LEN on endothelial network integrity. Within the effective range, although the presence of tumor cells inhibited T cell movement, LEN-treated CD8^+^ T cells exhibited enhanced migration in Transwell assays (Fig. 2, D and E) (*44*). In vasculature-free 3D tumor spheroids, LEN treatment of either T cells or tumor spheroids at 3 μM for 48 hours significantly increased T cell penetration (Fig. 2, F to I), indicating that LEN remodeled both vascular and cellular elements of the tumor microenvironment to promote T cell infiltration.

Quantitative polymerase chain reaction analysis and transcriptional profiling revealed that the enhanced infiltration was accompanied by coordinated activation of multiple adhesion and chemokine pathways (fig. S12). In T cells, LEN up-regulated *Vla4*, *Ccr7*, *Cxcr3*, and *Ccr5*, reinforcing migratory competence. In endothelial cells, LEN increased *Icam1*, *Vcam1*, and *Cd31*, enhancing adhesion and transmigration. Tumor cells responded by elevating *Ccl19*, *Ccl21*, *Cxcl9*, *Cxcl10*, and *Cxcl11*, generating chemokine gradients that attracted lymphocytes deep into the parenchyma (*45*, *46*). Together, these gene expression changes reshaped cytokine-chemokine-receptor networks among T cells, endothelial cells, and tumor cells, forming a transient chemokine-adhesion circuit that coupled vascular remodeling with immune infiltration (*47*–*51*).

The transient nature of LEN makes systemic, codelivery approaches unable to coordinate between vascular and immune responses. For example, intravenous administration of LEN failed to improve T cell infiltration in vivo, and intratumoral dosing induced only localized effects (fig. S13). In contrast, our T-LEN-nOPBP-1 conjugates significantly enhanced T cell penetration into 3D tumor spheroids by comparison with free LEN-nOPBP-1 and unconjugated controls (fig. S14). Whole-body fluorescence imaging revealed superior tumor localization of T-LEN-nOPBP-1 by comparison with free LEN-nOPBP-1 coadministered with T cells (Fig. 2, J and K). The results demonstrate that LEN-driven vascular remodeling and T cell infiltration can be effectively harnessed only when spatial and temporal coordination is achieved. The T-LEN-nOPBP-1 conjugate synchronizes the two processes together for effective vascular-immune modulation in solid tumors.

### LEN drives TCM differentiation and sustains effector activity

**Fig. 3.**
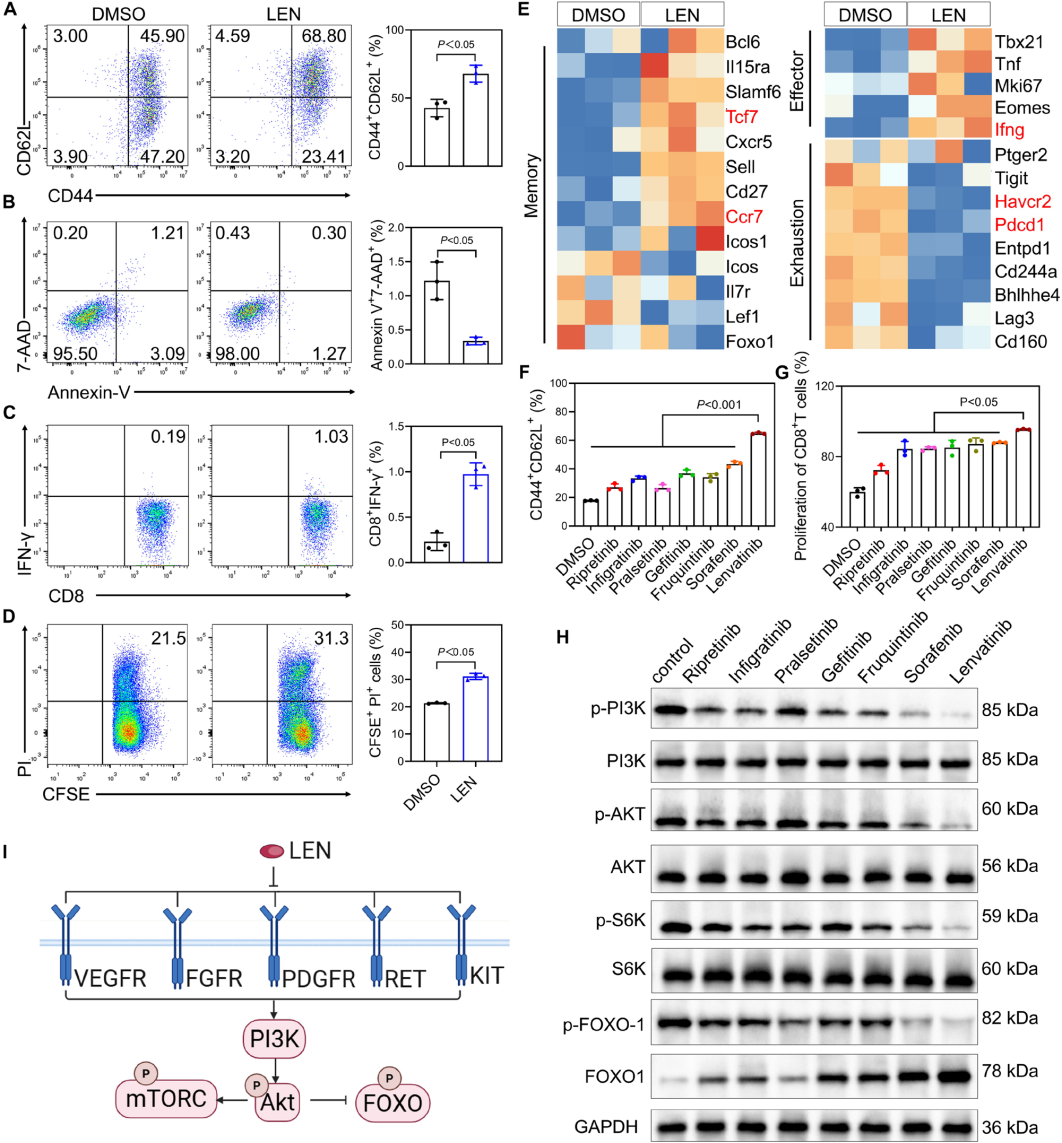
LEN promotes TCM differentiation through PI3K–AKT–mTOR-FOXO1 modulation. (**A**) Flow cytometry analysis of CD44 and CD62L expression on CD8^+^ T cells treated with 3 μM LEN for 72 hours, showing enhanced differentiation toward TCM. (**B**) Annexin V and 7-AAD staining of CD8^+^ T cells after 3-day LEN treatment, showing reduced late apoptosis. (**C**) Flow cytometry analysis of IFN-γ production in CD8^+^ T cells preactivated with anti-CD3 and anti-CD28 antibodies for 48 hours, followed by LEN treatment for 3 days. (**D**) B16-OVA tumor cells were prelabeled with CFSE and cocultured with preactivated OT-1–derived CD8^+^ T cells at an effector to target (E:T) ratio of 4:1 for 48 hours in the presence or absence of LEN. Tumor cell death was assessed by propidium iodide (PI) staining, with dead tumor cells defined as the CFSE^+^PI^+^ population and quantified by flow cytometry. (**E**) Transcriptomic profiling of LEN-treated CD8^+^ T cells showing up-regulation of memory-related (*Tcf7* and *Ccr7*) and effector-associated (*Ifng* and *Tnf*) genes, and down-regulation of exhaustion markers (*Havcr2*, *Pdcd1*, *Tigit*, and *Lag3*). (**F**) Comparative analysis of TCM frequency across TKIs under matched conditions, highlighting LEN as the most potent inducer. (**G**) Proliferative activity of CD8^+^ T cells treated with LEN assessed by CFSE dilution assay. T cell proliferation was quantified on the basis of the proportion of CFSE-diluted cells relative to the undivided population. Comparative analysis of CD8^+^ T cell proliferation across TKIs, showing LEN as the strongest enhancer. (**H**) Western blot analysis showing reduced phosphorylation of AKT and mTORC1 and activation of FOXO1 in LEN-treated T cells. (**I**) LEN targets VEGFR, FGFR, PDGFR, rearranged during transfection proto-oncogene (RET), and proto-oncogene c-Kit (KIT), converging on PI3K–AKT–mTOR suppression and FOXO1 activation to promote TCM differentiation. Data in (A), (B), (C), (D), (F), and (G) are mean values ± SD; *n* = 3.

Beyond vascular remodeling, LEN reprograms T cell fate and induces TCM formation. Treatment of tumor-infiltrating lymphocytes (TILs) from B16-OVA tumors with 1 or 3 μM LEN significantly expanded the TCM population (Fig. 3A), accompanied by increased expression of memory-associated markers TCF1 and CC-chemokine receptor 7 (CCR7) as assessed by flow cytometry (fig. S15), while reducing late apoptosis (Annexin V^+^/7-AAD^+^) (Fig. 3B), thereby supporting persistence within the tumor microenvironment. Moreover, LEN-treated T cells produced higher levels of interferon-γ (IFN-γ) (Fig. 3C and fig. S16), and exhibited stronger cytotoxicity against tumor cells by comparison with untreated controls (Fig. 3D). Transcriptomic profiling confirmed the TCM phenotype, showing up-regulation of memory- and effector-associated genes (*Tcf7*, *Ccr7*, *Ifng*, and *Tnf*) and down-regulation of exhaustion markers (*Havcr2*, *Pdcd1*, *Tigit*, and *Lag3*) (Fig. 3E). Furthermore, we compared LEN with a panel of TKIs of distinct selectivity. Several inhibitors increased TCM relative to dimethyl sulfoxide (DMSO) control (18%), including ripretinib (27%), pralsetinib (27%), infigratinib (33%), gefitinib (37%), fruquintinib (34%), and sorafenib (43%). In contrast, LEN produced the most pronounced effect, expanding TCM to 65% (Fig. 3F), and enhancing CD8^+^ T cell proliferation (Fig. 3G).

The AKT–mTOR signaling pathway is well established as a master regulator of T cell activation, proliferation, and metabolic reprogramming (*52*–*55*). FOXO1, a key downstream transducer of this pathway, directly transcriptionally controls genes essential for T cell memory differentiation and functional persistence (*56*, *57*). We therefore hypothesized that the extent of PI3K–AKT–mTOR pathway modulation, rather than inhibition of individual receptor tyrosine kinases, is associated with the induction of TCM. Consistent with this hypothesis, kinase inhibitors with distinct target spectra exerted minimal effects on total PI3K, AKT, and S6K protein levels, while variably increasing total FOXO1 expression. In contrast, all tested inhibitors were associated with reduced phosphorylation of PI3K, AKT, S6K, and FOXO1, whereas total protein levels remained largely unchanged, indicating modulation of pathway activation rather than changes in protein abundance. Notably, LEN was associated with the most pronounced reduction in AKT phosphorylation and downstream mTOR signaling, along with the lowest levels of FOXO1 phosphorylation, which correlated closely with its superior capacity to promote TCM differentiation (Fig. 3H). These observations are consistent with a model in which broader upstream RTK inhibition is linked to more effective attenuation of PI3K–AKT–mTOR signaling and preferential memory-associated T cell programming. A schematic summary of this signaling axis is shown in Fig. 3I.

In addition, we directly compared LEN with a canonical mTOR inhibitor (rapamycin) and a selective AKT inhibitor by flow cytometric analysis of CD44 and CD62L expression on mouse CD8^+^ T cells. As shown in the fig. S17, direct mTOR or AKT inhibition induced a more pronounced shift toward a TCM phenotype than LEN, consistent with their direct action on this pathway (*52*). While mTOR or AKT inhibition primarily modulates intrinsic T cell differentiation, it does not address tumor vasculature or synchronize T cell infiltration with vascular remodeling. In contrast, LEN promotes tumor vascular normalization, enhancing T cell infiltration into solid tumors, while also inducing moderate memory-associated phenotypic changes through partial suppression of the AKT-mTOR pathway. Thus, LEN provides a distinct biological profile that integrates vascular modulation with partial mTOR pathway attenuation, facilitating coordination of T cell infiltration and functional programming within solid tumors.

### T-LEN-nOPBP-1 enhances T cell programming, persistence, and antitumor efficacy

**Fig. 4.**
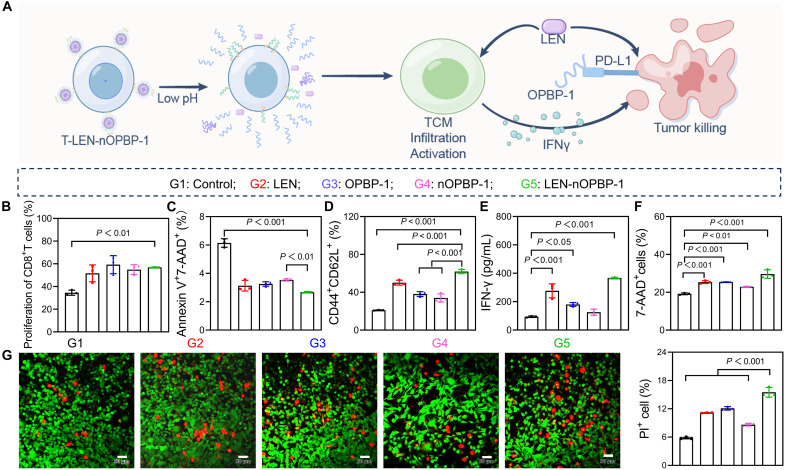
Immunomodulatory effects of the T-LEN-nOPBP-1 conjugates. (**A**) T-LEN-nOPBP-1 conjugates release LEN and the PD-L1 antagonistic peptide OPBP-1 in response to acidic tumor conditions, promoting TCM differentiation, immune activation, and tumor killing. (**B** to **D**) Functional analysis of CD8^+^ T cells after treatment with DMSO (G1, black), free LEN (G2, red), free OPBP-1 (G3, blue), T-nOPBP-1 (G4, pink), or T-LEN-nOPBP-1 (G5, green), including proliferation measured by CFSE dilution after 3 days (B), apoptosis determined by annexin V and 7-AAD staining (C), and memory phenotype profiling to determine TCM frequency after 5-day culture (D). (**E** to **G**) Effector functions of treated T cells cocultured with B16-OVA tumor cells, including IFN-γ secretion quantified by ELISA after 48 hours of coculture [effector-to-target ratio of 4:1 (E)], cytotoxicity evaluated by flow cytometric quantification of 7-AAD^+^ tumor cells (F), tumor cell death was determined by live/dead viability staining. Live tumor cells were identified by Calcein-AM staining (green), whereas dead tumor cells were identified by PI staining (red) (G). Scale bars, 50 μm. Data shown in (B) to (G) represent mean values ± SD; *n* = 3.

We evaluated the immunomodulatory effects of the T-LEN-nOPBP-1 conjugates by comparing T cells treated with DMSO (G1), free LEN (G2), free OPBP-1 (G3), T-nOPBP-1 (G4), or T-LEN-nOPBP-1 (G5) (Fig. 4A). LEN and OPBP-1 promoted T cell proliferation, reduced apoptosis, and increased TCM differentiation (Fig. 4, B to D). PD-L1 blockade alone also enhanced TCM formation using either free OPBP-1 or T-nOPBP-1, consistent with reports that PD-L1^+^ T cells suppress effector responses through the PD-L1–PD-1 axis (*58*, *59*). We observed substantial PD-L1 expression on activated CD8^+^ T cells as well as on tumor-infiltrating CD8^+^ T cells in the MC38-OVA model (fig. S18), suggesting an intrinsic inhibitory circuit that restrains T cell memory formation. Interrupting this pathway with OPBP-1 may partially alleviate such self-inhibitory signaling and be associated with a shift toward a memory phenotype.

The T-LEN-nOPBP-1 conjugates further enhanced both memory programming and effector activation, resulting in the highest TCM frequency among all groups. T-LEN-nOPBP-1–treated T cells secreted the greatest amounts of IFN-γ (Fig. 4E) and exhibited the strongest tumor-cell killing (Fig. 4, F and G) by comparison with LEN or OPBP-1 alone, confirming synergistic immune activation under acidic conditions that mimic the tumor microenvironments.

**Fig. 5.**
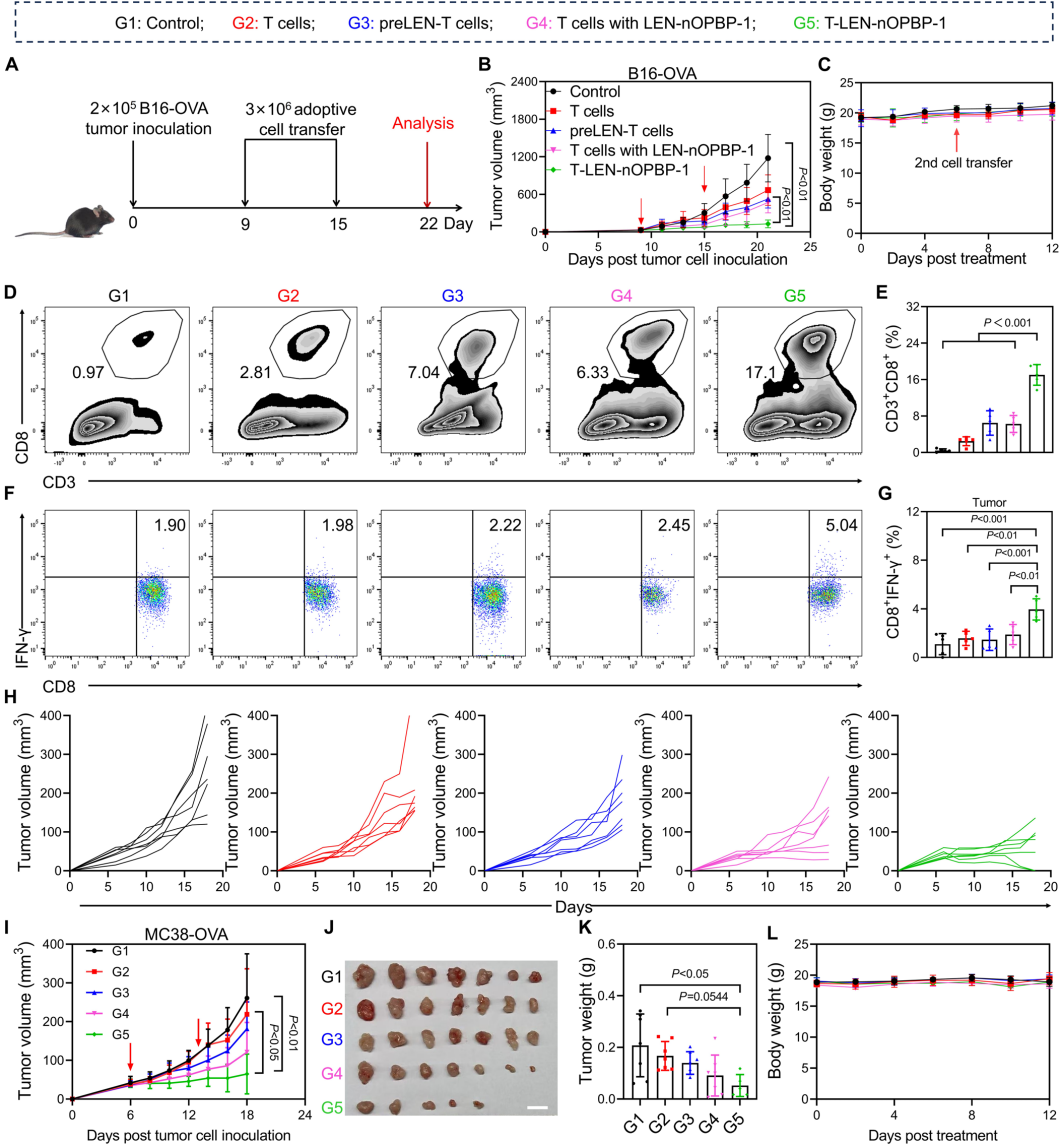
T-LEN-nOPBP-1 inhibits tumor growth in vivo. (**A**) B16-OVA melanoma model and the treatment protocol. (**B**) Tumor growth curves following treatment with saline (G1), OT-1 T cells (G2), LEN-pretreated T cells (G3), free LEN-nOPBP-1 micelles with T cells (G4), or T-LEN-nOPBP-1 conjugates (G5). (**C**) Body weight monitoring during treatment. (**D**) Representative flow cytometry plots showing CD3^+^CD8^+^ T cells among TILs. (**E**) Quantification of intratumoral CD3^+^CD8^+^ T cell frequencies from (D). (**F**) Flow cytometry analysis of CD8^+^IFN-γ^+^ T cells isolated from tumors following stimulation. (**G**) Quantification of CD8^+^IFN-γ^+^ T cells from (F). Data in (B), (C), (E), and (G) are mean values ± SD; *n* = 5 in B16-OVA model. (**H**) Individual tumor growth curves of MC38-OVA tumor model following two rounds of adoptive T cell transfer. (**I**) Tumor growth curves following treatment. (**J**) Images of excised tumors at the end of the experiment. (**K**) Quantification of tumor weights. (**L**) Body weight monitoring throughout treatment. Data in (I) and (K) are mean values ± SD; *n* = 7 in MC38-OVA tumor model.

We next assessed the therapeutic efficacy of the T-LEN-nOPBP-1 conjugates in vivo using subcutaneous B16-OVA melanoma and MC38-OVA colorectal tumor models in C57BL/6J mice (Fig. 5, A and H). Mice received saline (G1), OT-1 T cells (G2), LEN-pretreated T cells (G3), free LEN-nOPBP-1 micelles with T cells (G4), or T-LEN-nOPBP-1 conjugates (G5). Across both models, T-LEN-nOPBP-1 achieved the most pronounced tumor inhibition, significantly outperforming free T cells or unconjugated micelles (Fig. 5, B and I to K), while maintaining stable body weight (Fig. 5, C and L). Tracking of Cy5-labeled CD8^+^ T cells revealed markedly greater accumulation of T-LEN-nOPBP-1 conjugates in key immune organs and tumors relative to free T cells or those codelivered with LEN-nOPBP-1 micelles (fig. S19). Immunofluorescence analysis of B16-OVA tumor sections revealed that treatment with the T cell–nanodrug conjugate markedly increased intratumoral CD8^+^ T cell infiltration and promoted more organized and continuous CD31^+^ vascular structures compared with control groups. The spatial association between normalized vasculature and enhanced CD8^+^ T cell penetration provides in vivo evidence that coordinated vascular remodeling facilitates T cell entry into tumor tissue (fig. S20). The results indicate that direct nanodrug conjugation prolongs T cell survival and systemic retention, establishing a foundation for durable therapeutic activity. Flow cytometric analysis further demonstrated that tumors treated with T-LEN-nOPBP-1 contained a substantially higher proportion of CD3^+^CD8^+^ TILs than any other group (Fig. 5, D and E), whereas free T cells, with or without LEN-nOPBP-1, failed to achieve a similar level of infiltration. Moreover, T-LEN-nOPBP-1 treatment significantly increased the proportion of CD8^+^Ki67^+^ T cells in tumors (fig. S21). In addition, CD8^+^IFN-γ^+^ T cells were elevated in tumors (Fig. 5, F and G), draining lymph nodes (fig. S22, A and B), and spleens (fig. S22, C and D), indicating reinforced systemic effector activation. Notably, T-LEN-nOPBP-1 also increased the proportion of TCM (CD44^+^CD62L^+^) among splenic CD8^+^ T cells by sixfold relative to free T cells (fig. S22, E and F), showing enhanced memory differentiation and sustained immune potential.

Having established potent immune activation in the B16-OVA melanoma model, we next applied T-LEN-nOPBP-1 to a subcutaneous MC38-OVA colorectal carcinoma model (Fig. 5H). Following two rounds of adoptive therapy, T-LEN-nOPBP-1 inhibited tumor growth by comparison with all controls (Fig. 5, I to K), including mice receiving LEN-pretreated T cells or free LEN-nOPBP-1 micelles. Notably, complete tumor regression occurred in two of seven mice treated with T-LEN-nOPBP-1, demonstrating its curative potential. Body weight remained stable throughout the treatment (Fig. 5L), and hematoxylin-eosin staining of major organs revealed no evident histopathological abnormalities (fig. S23), confirming systemic tolerability. Consistent with observations in the B16-OVA model, T-LEN-nOPBP-1 enhanced CD3^+^CD8^+^ T cell infiltration (fig. S24, A and B), increased IFN-γ production (figs. S24, C and D, and S25, A and B), and expanded TCM populations in draining lymph nodes, spleens, and tumors (fig. S25, C to E), suggesting that TCMs preferentially reside in or recirculate through secondary lymphoid organs (*60*, *61*).

## DISCUSSION

ACT for solid tumors is limited by abnormal vasculature that blocks infiltration and defective immune persistence within the suppressive tumor microenvironment (*62*, *63*). LEN can transiently normalize vessels, but the brief pharmacologic window restricts T cell trafficking (*40*, *64*). Meanwhile, the transferred T cells rapidly lose proliferative capacity and functional fitness during tumor infiltration, leading to exhaustion and eventual relapse. The resulting temporal and spatial discordance between vascular remodeling and T cell activation has remained a central obstacle to durable ACT efficacy.

We addressed the challenge by identifying LEN as a dual vascular-immune modulator and by creating a tumor-responsive T cell–nanodrug conjugate (T-LEN-nOPBP-1). We showed that LEN promoted TCM differentiation in addition to its capability to normalize tumor vessels. The immunoregulatory effect correlates with attenuation of RTK-driven PI3K–AKT–mTOR signaling and reduced FOXO1 phosphorylation, thereby preserving FOXO1-dependent memory-associated transcriptional programs. Further, the conjugate composition aligns the functions of LEN with T cell infiltration in the tumor microenvironment. Each T cell carries its own pharmacologic payload, releasing LEN and OPBP-1 only in the acidic tumor milieu to align vascular remodeling, infiltration, and immune activation. Through bioorthogonal conjugation and tumor-specific release (*65*–*68*), T-LEN-nOPBP-1 provides a spatiotemporally controlled immunotherapy approach, in which T cells both execute and orchestrate therapy, transforming transient drug signals into sustained antitumor immunity.

Future studies should focus on enhancing the long-term therapeutic persistence of T cell–conjugated strategies. The chemically conjugated payloads are non-heritable and would progressively dilute during antigen-driven clonal expansion, resulting in a finite duration of activity. Staged supplementation of nanodrug conjugates may leverage residual surface azide functionalities to enable additional micelle attachment and prolong localized drug delivery in a controllable, nongenetic manner. The approach of using T cells as self-coordinating delivery units can extend to agents that modulate metabolism, epigenetic plasticity, or stromal signaling.

## MATERIALS AND METHODS

### Cell lines

MC38-OVA cells were provided by C. Lu (Shenzhen Bay Laboratory, China). CHO-K1 cells stably expressing murine PD-L1 (CHO-K1–mPD-L1) were generated via lentiviral transduction with pLVX-Puro. B16-OVA, CHO-K1, and CHO-K1–mPD-L1 cells were maintained in RPMI-1640 medium (Gibco, USA) supplemented with 10% fetal bovine serum (Sigma-Aldrich, USA), penicillin (100 U/ml), and streptomycin (100 μg/ml; Solarbio, China) in a humidified incubator at 37°C with 5% CO_2_.

### Mice

C57BL/6J mice were maintained in the specific pathogen–free facility at Sun Yat-sen University. OT-1 mice (OVA_257–264_ TCR transgenic mice) were provided by X. Yang (Shanghai Jiao Tong University, China). Animal experimental procedures were carried out in accordance with national and institutional guidelines and were approved by the Ethics Committee of Sun Yat-sen University (SYSU-YXYSZ20230605) (*30*).

### Micelle preparation and characterization

PCL–PEG–DBCO (1 μM) and PCL–OPBP-1 (16 μM) were mixed at a molar ratio of 1:16 and codissolved with LEN (4.8 μM). The mixture was slowly added to saline under magnetic stirring for 5 min, followed by sonication for 12 min to generate acid-responsive LEN-nOPBP-1 micelles. Particle size, zeta potential, and colloidal stability were measured by dynamic light scattering (ZS90, Malvern Analytical, UK), and morphology was examined by transmission electron microscopy (H-7650, Hitachi, Japan). Drug loading efficiency, encapsulation rate, and pH-responsive release kinetics of LEN were determined using a UV-Vis spectrophotometer (Tianmei, China) and a fluorescence spectrophotometer (PerkinElmer, USA).

### PD-L1 blocking assay

CHO-K1 cells stably overexpressing PD-L1 (CHO-K1–mPD-L1) were harvested at 3 × 10^5^ cells/tube. OPBP-1 peptide or nOPBP-1 micelles (in defined ratios) were preincubated with 50 ng of PD-L1–Fc (Sino Biological) on ice for 30 min to form competitive complexes. The complexes were then incubated with the cell suspension on ice for an additional 30 min, followed by staining with an anti-Fc antibody (eBioscience) for 30 min in the dark. PD-L1 cell-surface occupancy was analyzed by flow cytometry (BD FACSCanto II, BD Biosciences).

### T cell conjugation

Jurkat cells were cocultured with 50 μM acetylated *N*-azidoacetylmannosamine (Ac4ManNAz) (New Research Biosciences, Shanxi, China) for 3 days to obtain azide-modified T cells. These cells were then reacted with fluorescein isothiocyanate (FITC)–nOPBP-1 micelles at 37°C for 1 hour to enable covalent conjugation via bioorthogonal chemistry. Binding efficiency and nanoparticle stability on the T cell surface were evaluated by flow cytometry, confocal fluorescence microscopy (Olympus FV30000), and scanning electron microscopy (Phenom World B.V.).

### Tube formation assay

To assess LEN-induced vascular normalization, human umbilical vein endothelial cells (HUVECs; 4 × 10^5^ cells/ml) were seeded onto Matrigel (20 μl per well, precooled 24-well plates) and treated with LEN (0.3 to 30 μM) and VEGF165 (50 ng/ml, Dakewe) (*69*, *70*). Each well received 200 μl of cell suspension and 200 μl of drug-containing medium. Tube formation was imaged at multiple time points (2 to 24 hours) using an inverted microscope, and branching points were quantified in three random fields using ImageJ (version 1.53).

### Alginate-encapsulated angiogenesis model

B16-OVA cells (1.0 × 10^7^ cells/ml) were suspended in 1.5% (w/v) sodium alginate and dripped into 80 mM CaCl_2_ under stirring to form ∼2-mm encapsulated spheres, solidified at room temperature for 30 min (*71*). Two tumor alginate beads were implanted subcutaneously into the dorsal flank of C57BL/6 mice under chloral hydrate anesthesia. Fourteen days later, LEN (0, 0.3, 1, or 3 mg/kg) was injected locally around the implants. At 6 and 12 hours postinjection, the spheres were excised, and surface vascularization was assessed microscopically.

### Transwell migration assay

Mouse-derived T cells were activated with IL-2 (100 U/ml), anti-CD3 (1 μg/ml), and anti-CD28 (1 μg/ml) for 72 hours (*72*). A total of 2 × 10^5^ T cells (1 × 10^6^ cells/ml, 200 μl) were added to the upper chamber of a 5-μm transwell insert. The lower chamber contained 400 μl of complete medium with DMSO or varying concentrations of LEN. For coculture conditions, 2 × 10^5^ B16-OVA cells were seeded in the lower chamber. After 48 hours of incubation at 37°C, migrated CD8^+^ T cells were quantified by flow cytometry.

### 3D tumor spheroid infiltration assay

B16-OVA cells (8000 cells per well) were seeded into agarose-coated 96-well plates and centrifuged at 2000*g* for 10 min, followed by 5-day incubation to form spheroids. On day 5, spheroids were cocultured with carboxyfluorescein diacetate succinimidyl ester (CFSE)-labeled T cells (1 × 10^6^ cells per well) that had been pretreated with DMSO or LEN for 24 hours. After 48 hours, T cell infiltration was analyzed by confocal microscopy (Olympus FV1200). For tumor cell–directed pretreatment, LEN was applied to spheroids 24 hours before T cell coculture under otherwise identical conditions.

### qRT-PCR analysis

T cells, HUVECs, and B16-OVA cells (1 × 10^6^ cells per group) were treated with 3 μM LEN for 72 hours. Total RNA was extracted using TRIzol and reverse-transcribed with PrimeScript RT Master Mix. Quantitative polymerase chain reaction (qPCR) was performed with SYBR Green Supermix (Bio-Rad) under the following cycling conditions: 95°C for 3 min; 40 cycles of 95°C for 10 s and 60°C for 30 s. Gene expression (*Ccr7*, *Cxcr3*, *Vla-4*, and *Tsp-1*) was normalized to glyceraldehyde-3-phosphate dehydrogenase and β-actin.

### In vivo distribution post T cell transfer

OT-1 CD8^+^ T cells were labeled with DBCO-Cy5 (*73*) and transferred (1 × 10^6^ per mouse) into B16-OVA tumor–bearing mice (*n* = 4 per group) treated as follows: (i) saline control, (ii) T cells only, (iii) T cells + LEN (1 mg/kg, i.v.), and (iv) T cells + LEN (1 mg/kg, subcutaneously near tumor). Twelve hours postinfusion, major organs were harvested, and T cell biodistribution was analyzed using an in vivo imaging system.

### T cell phenotype and function assays

T cells isolated from mouse spleens and lymph nodes were prepared as a single-cell suspension (1 × 10^6^ cells/ml), and 200 μl per well was seeded in round-bottom 96-well plates. Cells were stimulated with IL-2 (100 U/ml, PeproTech), anti-CD3 (1 μg/ml, clone 145-2C11, BioLegend), and anti-CD28 (1 μg/ml, clone 37.51, BioLegend), in the presence of different drugs (ripretinib, infigratinib, pralsetinib, gefitinib, fruquintinib, sorafenib, and LEN) at 3 μM, and incubated at 37°C for 72 hours. For memory phenotype analysis, cells were stained with anti–CD8-PE (clone 53-6.7), anti–CD44-APC (clone IM7), and anti–CD62L-PE-Cyanine7 (clone MEL-14) (all from eBioscience), and the proportion of TCMs (CD44^+^CD62L^+^) was analyzed by flow cytometry (*74*). For intracellular cytokine detection, cells were treated with brefeldin A for 4 hours to block exocytosis, followed by fixation and staining with anti–IFN-γ–PE (clone XMG1.2, eBioscience). IFN-γ^+^ T cells were quantified by flow cytometry. In parallel, IFN-γ secretion was measured by enzyme-linked immunosorbent assay (ELISA) in culture supernatants from CD8^+^ T cells preactivated with anti-CD3 and anti-CD28 antibodies for 48 hours and then treated with LEN for 3 days. For cytotoxicity assays, B16-OVA tumor cells were prelabeled with CFSE and cocultured with preactivated OT-1–derived CD8^+^ T cells at an effector to target (E:T) ratio of 4:1 for 48 hours in the presence or absence of LEN. Tumor cell death was assessed by propidium iodide (PI) staining, with dead tumor cells defined as the CFSE^+^PI^+^ population and quantified by flow cytometry.

### Western blotting

T cells were treated with 3 μM of different drugs (ripretinib, infigratinib, pralsetinib, gefitinib, fruquintinib, sorafenib, and LEN) for 72 hours, then lysed with RIPA lysis buffer (Thermo Fisher Scientific, Waltham, MA, USA) (containing protease/phosphatase inhibitors), and centrifuged at 12,000*g* for 15 min to collect the supernatant. Proteins from the supernatant were resolved by 10% SDS–polyacrylamide gel electrophoresis, transferred to polyvinylidene difluoride membranes, and blocked with 5% nonfat milk for 1 hour. Membranes were incubated overnight at 4°C with primary antibodies against PI3K (TA3242, Abmart), AKT (T55561, Abmart), S6K (T55546, Abmart), and FOXO1 (C29H4, CST), or their phosphorylated forms: p-PI3K (T40064, Abmart), p-AKT (66444-1-Ig, Abmart), p-S6K (14485-1-AP, Abmart), and p-FOXO1 (E1F7T, CST), all at 1:1000. After incubation with horseradish peroxidase–conjugated secondary antibodies (1:5000) for 1 hour at room temperature, bands were visualized using enhanced chemiluminescence detection.

### Transcriptome analysis

RNA sequencing libraries were prepared and sequenced on the Illumina NovaSeq 6000 platform, generating 150 bp paired-end reads with ∼55 million raw reads per sample. After quality control with fastp, ∼49 million clean reads per sample were retained and aligned to the reference genome using HISAT2. Expression levels were quantified as fragments per kilobase of transcript per million mapped reads, and read counts were obtained using HTSeq-count. Principal components analysis was performed in R (version 3.2.0) to assess sample clustering. Differential gene expression was analyzed using DESeq2, with *Q* values <0.05 and fold changes>2 or <0.5 as thresholds. DEGs were visualized via hierarchical clustering, radar plots (top 30 genes), and enrichment analysis for gene ontology, Kyoto Encyclopedia of Genes and Genomes, Reactome, and WikiPathways terms, all performed in R (version 3.2.0). Gene set enrichment analysis (GSEA) was conducted using the GSEA software based on predefined gene sets.

### Functional assessment of T-LEN-nOPBP-1 conjugates

The effects of LEN-nOPBP-1 conjugation on T cell proliferation, apoptosis, differentiation, function, and 3D cell spheroid infiltration ability were assessed using CFSE staining (eBioscience), immunophenotyping, flow cytometry, and confocal fluorescence microscopy.

### In vivo biodistribution of T-LEN-nOPBP-1 conjugates

CD8^+^ T cells isolated from OT-1 mouse spleens and lymph nodes were metabolically labeled with azido-mannose and fluorescently tagged using DBCO-Cy5. B16-OVA tumor–bearing C57BL/6 mice were randomized into three groups (*n* = 3 per group): (i) saline, (ii) T cells plus free micelles (intravenous administration of LEN micelles followed by T cell transfer), and (iii) T-LEN-nOPBP-1 conjugates. Each mouse received 1 × 10^6^ T cells via tail vein injection. Twelve hours post-transfer, major organs were harvested and analyzed for fluorescence distribution using a small animal imaging system.

### In vivo antitumor efficacy

B16-OVA (2 × 10^5^ cells per mouse) and MC38-OVA (1 × 10^6^ cells per mouse) cells were subcutaneously injected into the right flank of 5- to 8-week-old C57BL/6J mice. When tumors reached 30 to 40 mm^3^ with uniform size distribution, mice were randomized using an S-curve allocation and assigned to five treatment groups (*n* = 5 mice per group in the B16-OVA tumor model and *n* = 7 mice per group in the MC38-OVA tumor model): (i) saline, (ii) T cells, (iii) preLEN-T cells (T cells pretreated with free LEN), (iv) LEN-nOPBP-1 + T cells (administered separately), and (v) T-LEN-nOPBP-1 conjugates. Each mouse received two tail vein injections of 3 × 10^6^ T cells (on days 9 and 15 for B16-OVA, and on days 6 and 13 for MC38-OVA). Tumor volume, body weight, and survival were monitored daily. At the end point, tumors were excised, enzymatically digested with collagenase and deoxyribonuclease I (Sigma-Aldrich, USA) for 40 min at 37°C, and filtered through a 100-μm cell strainer to obtain single-cell suspensions. Tumor tissues were dissected, digested, and processed for single-cell suspensions to evaluate CD8^+^ T cell infiltration using flow cytometry. To determine the intracellular IFN-γ and Ki67 of CD8^+^ T cells from tumor tissue, lymphocytes were separated by Percoll (GE Healthcare, USA). The isolated lymphocytes were carefully collected, washed twice with PBS (pH 7.2), and plated in a 24-well plate (1 × 10^6^ cells per well). After adding 1 μl of protein transport inhibitor cocktail (containing brefeldin A, BD, USA), the cells were stimulated by OVA peptide (1 μg/ml) for 6 hours. Cells were then collected and stained for surface markers including CD3 (17A2), CD8α (53-6.7), CD44, and CD62L (MEL-14), followed by intracellular staining for IFN-γ (XMG1.2) and Ki67 (SolA15). Spleens and tumor-draining lymph nodes were harvested in parallel and processed into single-cell suspensions by mechanical dissociation and red blood cell lysis. Cells from these tissues were subjected to the same ex vivo antigen-specific restimulation, surface, and intracellular staining procedures, allowing direct comparison of CD8^+^ T cell phenotypes and functional states across tumor and peripheral lymphoid compartments (*30*).

### Statistical analysis

Statistical analyses were performed using GraphPad Prism 8.0.2. For comparisons between two groups, a one-sided Mann-Whitney test was used. For comparisons among multiple groups, one-way analysis of variance (ANOVA) followed by Tukey’s multiple comparisons test was applied. Data are presented as mean ± SD. *P* < 0.05 was considered significant. *P* < 0.01 and *P* < 0.001 were considered highly significant.

## Supplementary Materials

### This PDF file includes:

Supplementary Note Figs. S1 to S25
