# Supplementary material for: T cell–nanodrug conjugates synchronize vascular normalization and immune activation for solid tumor therapy
Source: Sci Adv. 2026 Aug 7;12(32):eadp2955. doi: 10.1126/sciadv.adp2955 (PMC13450223; doi:10.1126/sciadv.adp2955)
Supplement: Supplementary file 1 [file sciadv.adp2955_sm.pdf]

Science Advances
AAAS

Supplementary Materials for

T cell–nanodrug conjugates synchronize vascular normalization and immune activation for solid tumor therapy

Xin Yang *et al.*

Corresponding author: Yujia Zhang, yujia.zhang@epfl.ch; Dalin Wu, wudlin6@mail.sysu.edu.cn; Juan Liu, liuj756@mail.sysu.edu.cn

Sci. Adv. **12**, eadp2955 (2026)
DOI: 10.1126/sciadv.adp2955

This PDF file includes:

Supplementary Note
Figs. S1 to S25

Supplementary Note

Synthesis and Characterization of PCL-PEG-DBCO and PCL-OPBP-1

The micelles self-assemble from two three-armed PCL-based amphiphiles bearing acid-labile β-thiopropionate linkages—one presenting the PD-L1–blocking peptide OPBP-1 and the other providing dibenzocyclooctyne (DBCO) groups for bioorthogonal conjugation. The synthesis procedures and structural elucidation of dibenzocyclooctyne-functionalized three-armed poly (ε-caprolactone)–poly (ethylene glycol) (PCL-PEG-DBCO) and three-armed poly (ε-caprolactone) conjugating with PD-L1 blocking peptide OPBP-1 (PCL-OPBP-1) are illustrated in Scheme 1.

Acrylate-terminated three-arm PCL (PCL-acrylate) was synthesized by reacting PCL-triol (984 mg, 0.492 mmol) with acrylic acid (177 mg, 2.460 mmol), DMAP (12 mg, 0.067 mmol), and DCC (202 mg, 0.979 mmol) in 10 mL dry dichloromethane under argon at room temperature for 24 h. The product was precipitated with precooled diethyl ether, recrystallized twice, dialyzed (MWCO: 1000 Da) for 3 days, and vacuum-dried to yield a white powder. Structure was confirmed by ^1H NMR (600 MHz, Bruker, USA).

To prepare PCL-PEG-DBCO, acrylate-terminated PCL (206.8 mg, 0.094 mmol) was reacted with HS-PEG-DBCO (850 mg, 0.425 mmol) in dry DMSO (20 mL) in the presence of AIBN (9.25 mg, 0.056 mmol). The mixture was degassed with argon for 15 min and stirred at 70 °C overnight. The crude product was precipitated in diethyl ether, recrystallized twice, and dialyzed (MWCO: 1000 Da) in deionized water for 72 h before lyophilization. Product identity was confirmed by ^1H NMR, and molar mass and dispersity were analyzed by GPC (Waters e2695, DMSO as eluent). PCL-OPBP-1 was synthesized using the same procedure, substituting OPBP-1 peptide for HS-PEG-DBCO.

The successful conjugation was confirmed by 1H NMR spectroscopy. Specifically, the complete disappearance of the characteristic vinyl proton signals (δ = 5.80–6.45 ppm) corresponding to the acrylate groups on PCL (Fig. S1–S3, Supporting Information) indicates the quantitative consumption of the C=C double bonds during the thiol-ene reaction. Additionally, the appearance of new aromatic proton peaks in the regions of 7.09–7.40 ppm, 7.90–8.41 ppm, and 6.73–7.45 ppm (Fig. S3 and Fig. S4) further supports the successful introduction of the DBCO and OPBP moieties. These peaks are attributable to the aromatic protons of DBCO as well as the histidine, tyrosine, and phenylalanine residues within the OPBP segment. GPC analysis revealed

that PCL-PEG-DBCO and PCL-OPBP-1 exhibit number-average molecular weights (M_n) of approximately 13,000 and 11,000, with corresponding PDI values of 1.31 and 1.15, respectively.

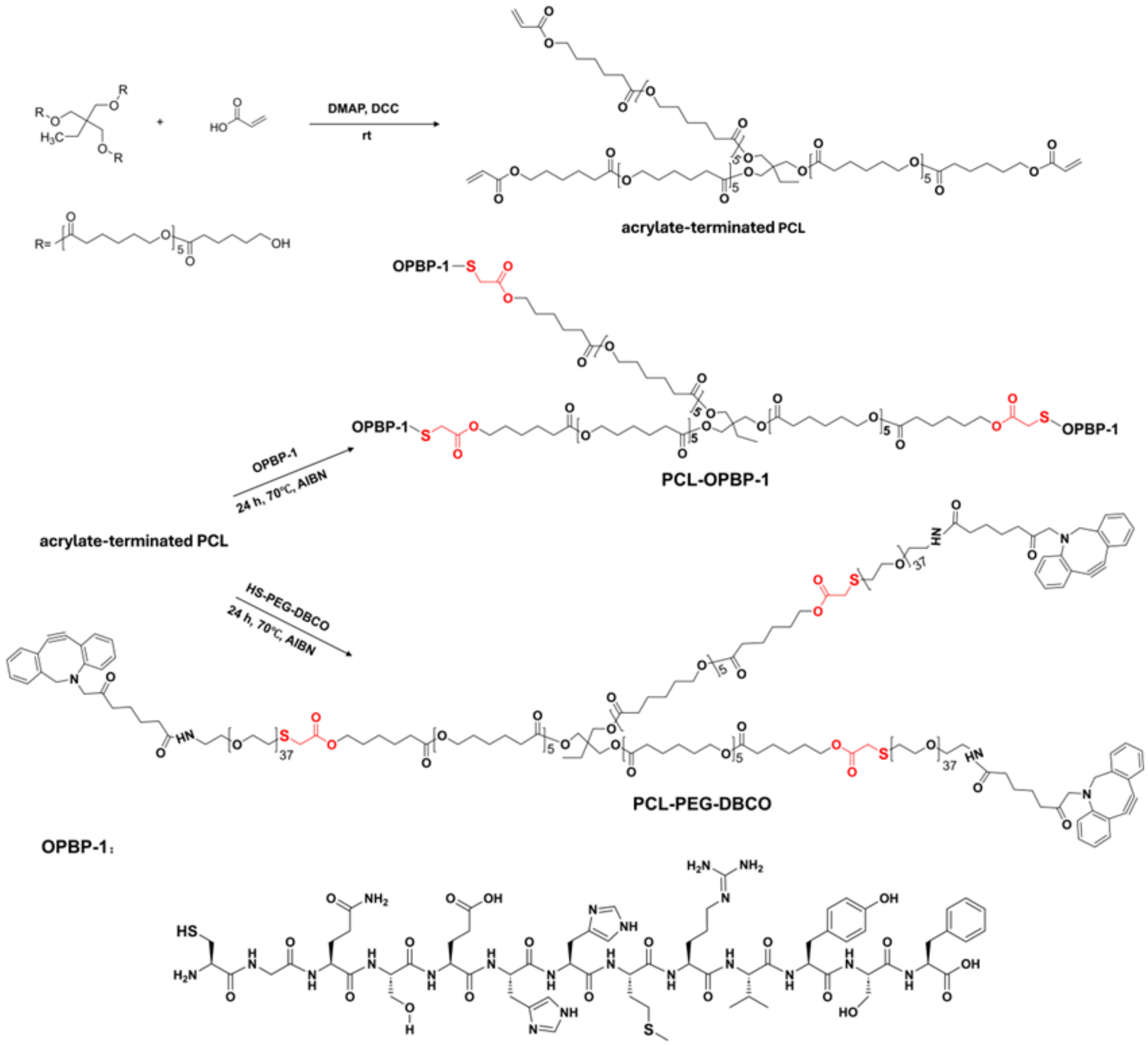

Fig. S1. Synthesis of PCL-PEG-DBCO and PCL-OPBP-1.

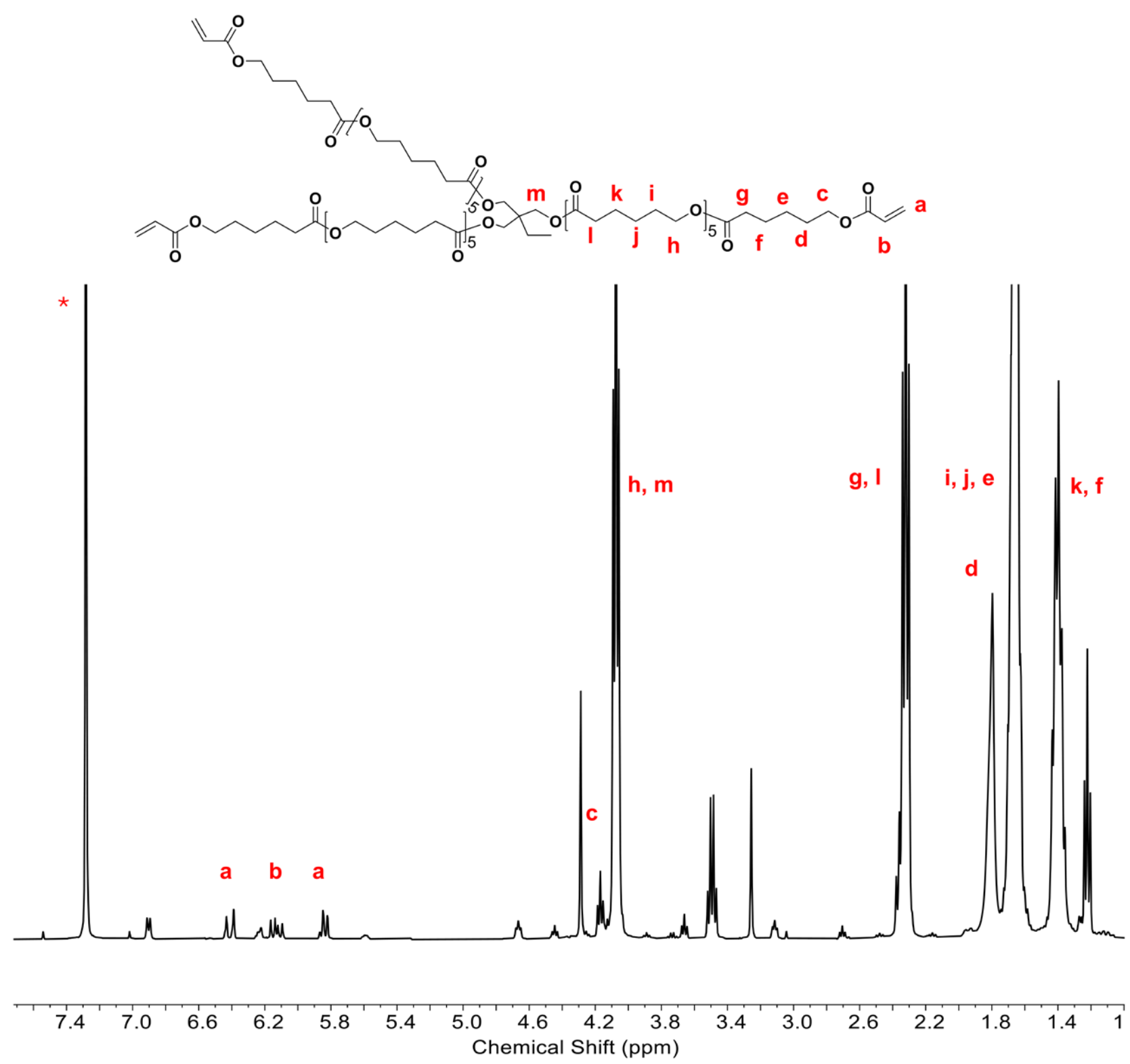

Fig. S2. ^{1}H NMR spectrum of acrylate-terminated PCL.

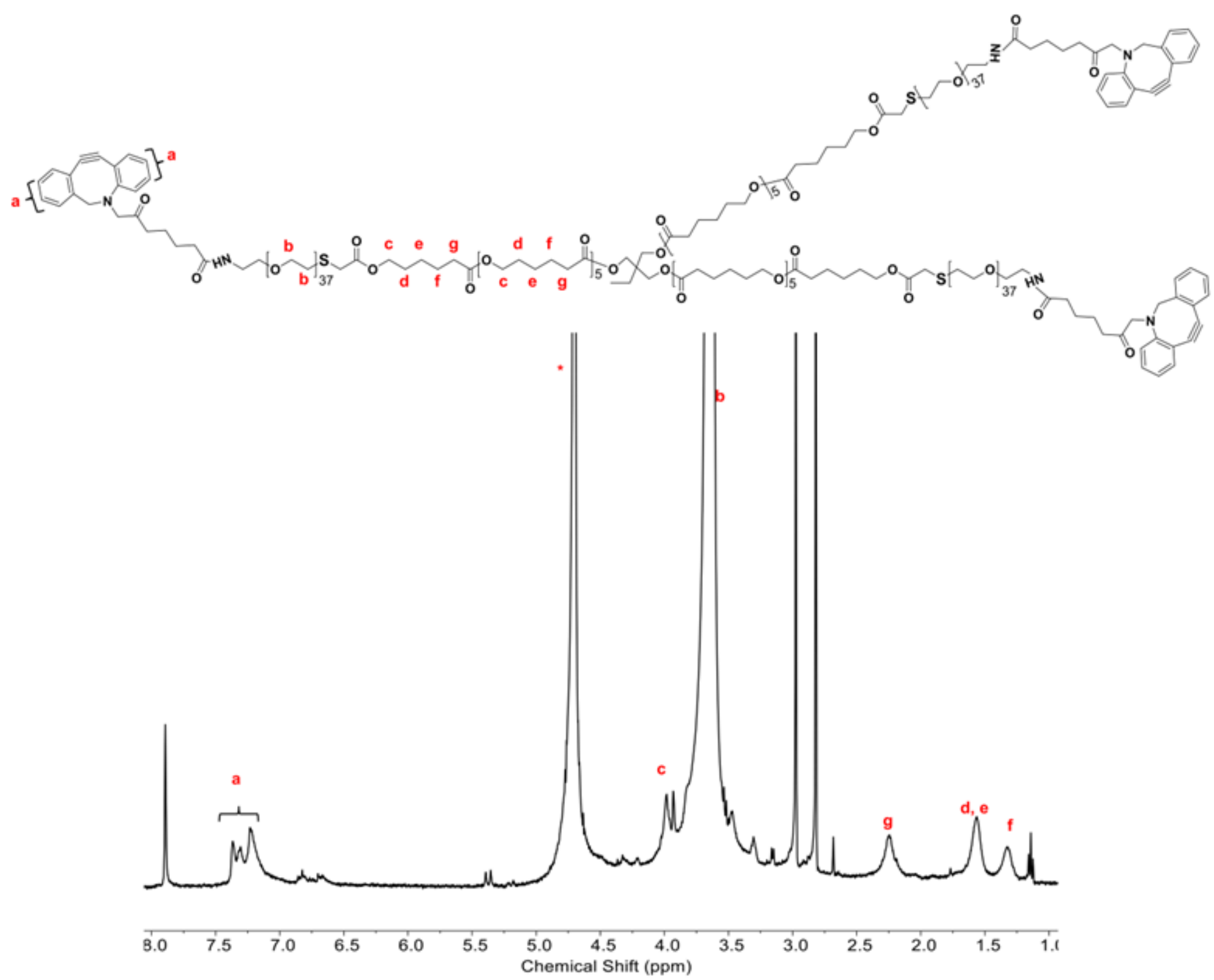

Fig. S3. ^{1}H NMR spectrum of PCL-PEG-DBCO.

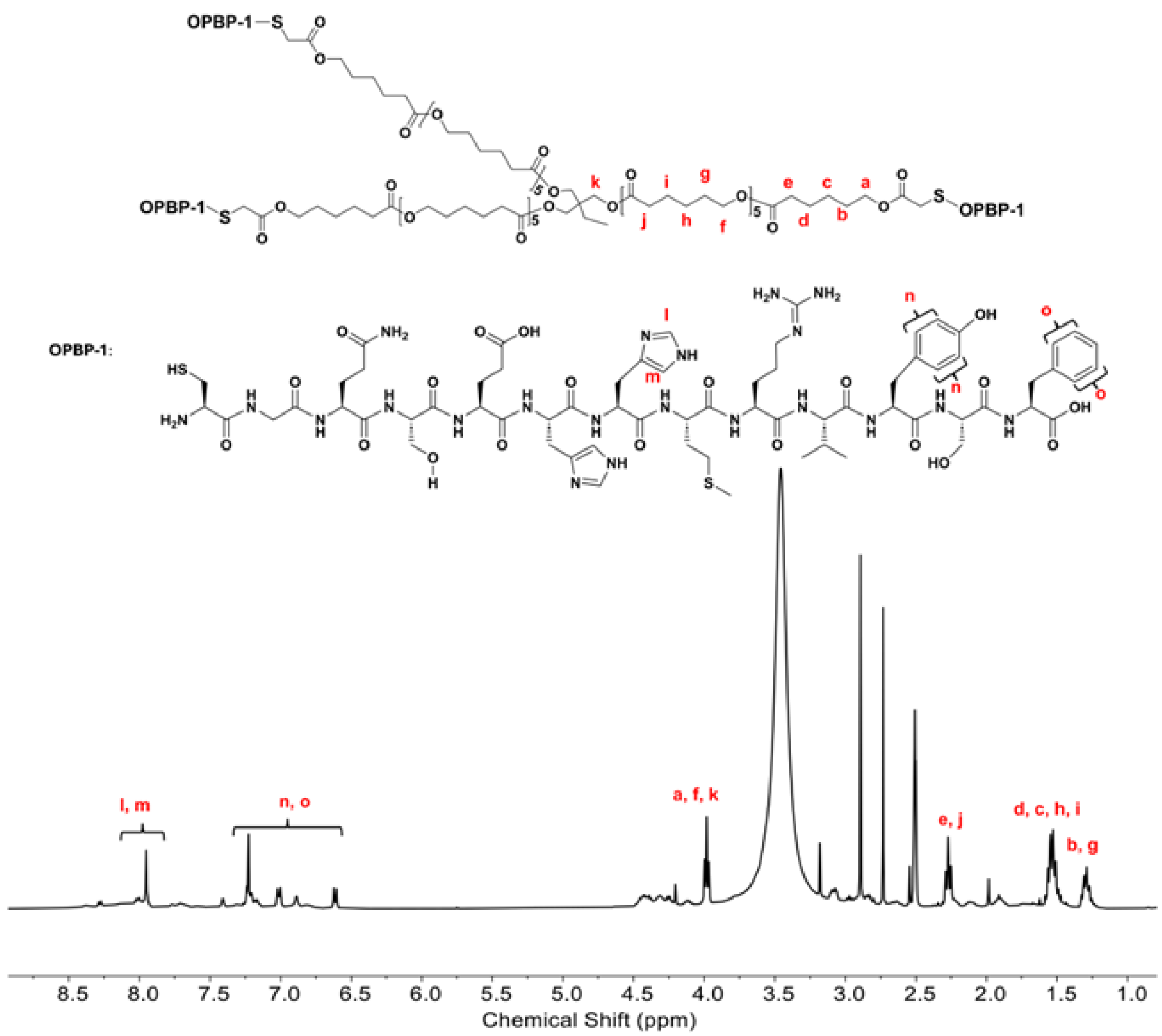

Fig. S4. ^{1}H NMR spectrum of PCL-OPBP-1.

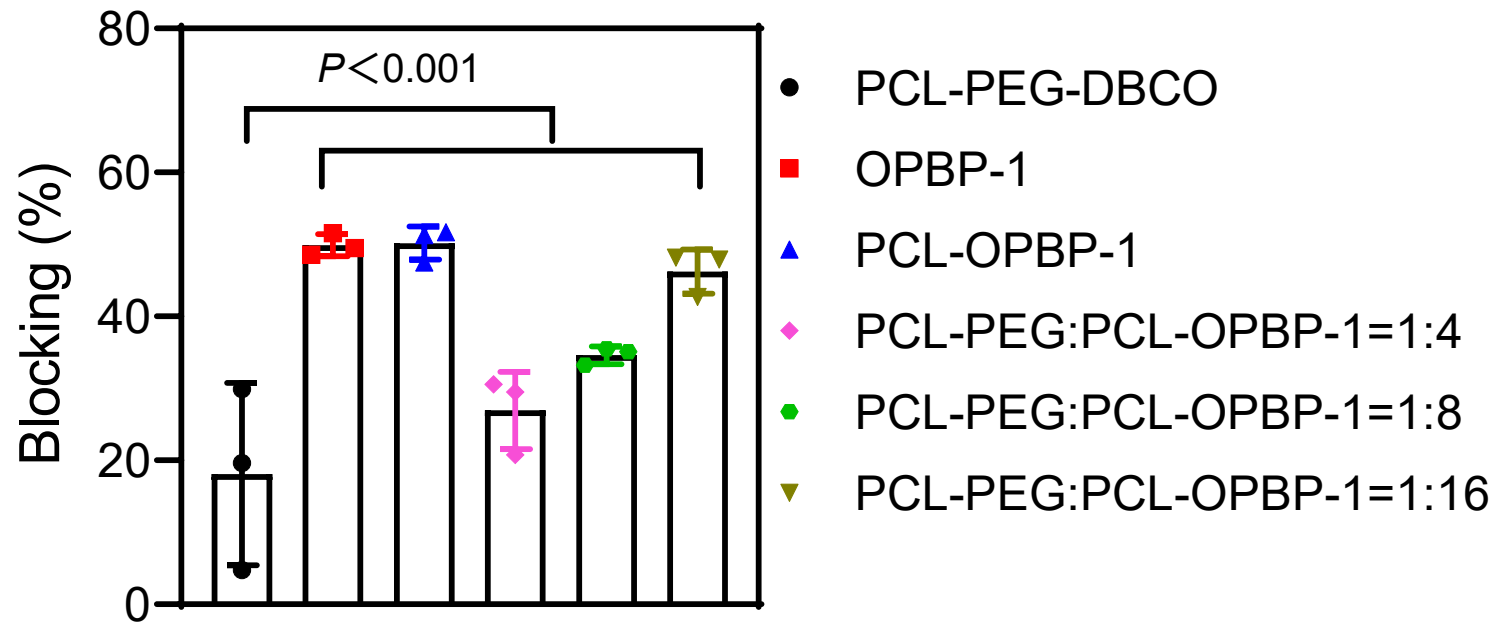

Fig. S5. Assessment of PD-1/PD-L1 blockade efficiency across different micelle formulations. Blocking activity was quantified by measuring the reduction in fluorescence intensity of PD-L1 binding to PD-1 in treated samples relative to untreated controls. Values are means ± SD; n = 3.

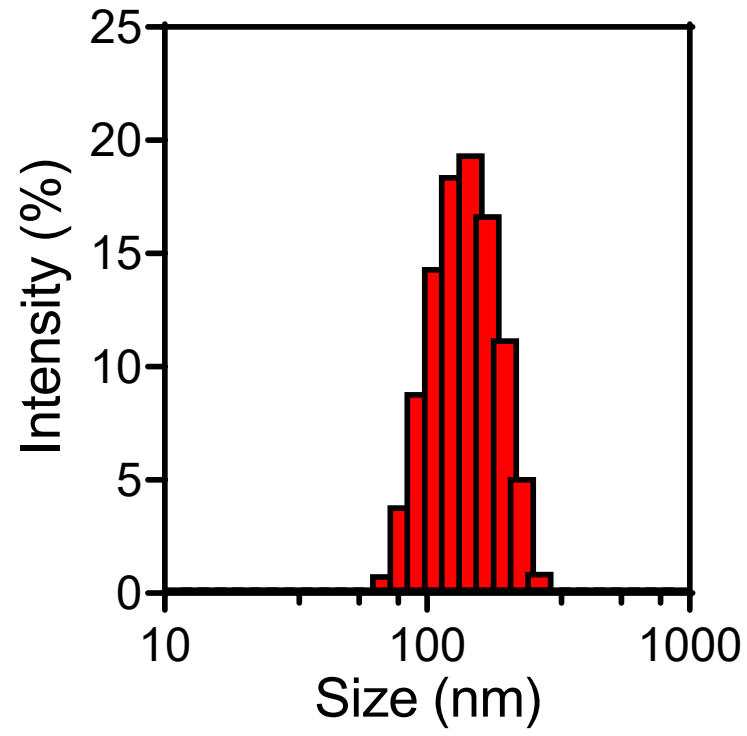

Fig. S6. DLS analysis indicated a hydrodynamic diameter of 171 ± 26 nm.

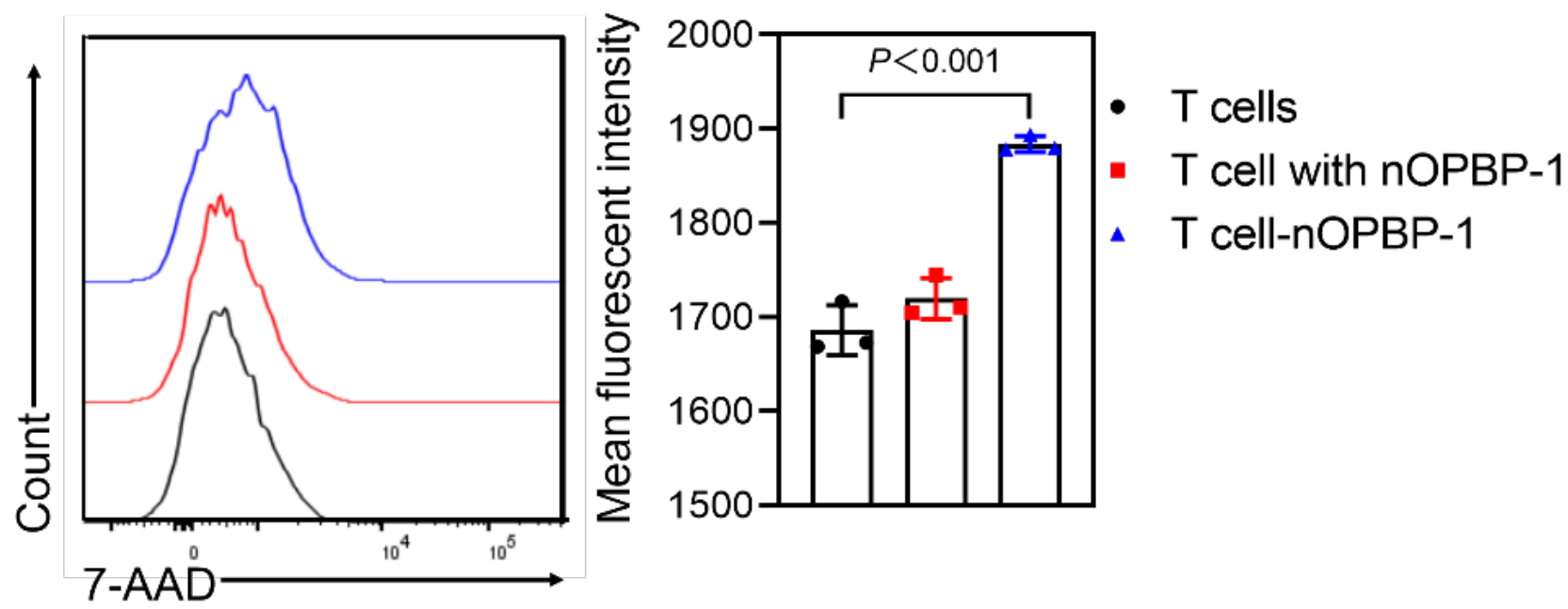

Fig. S7. Flow cytometry analysis of micelle-T cell conjugation. Shown are T cells, unlabeled T cells with nOPBP-1, and azide-labeled T cells with LEN-nOPBP-1. Values are means ± SD; n = 3.

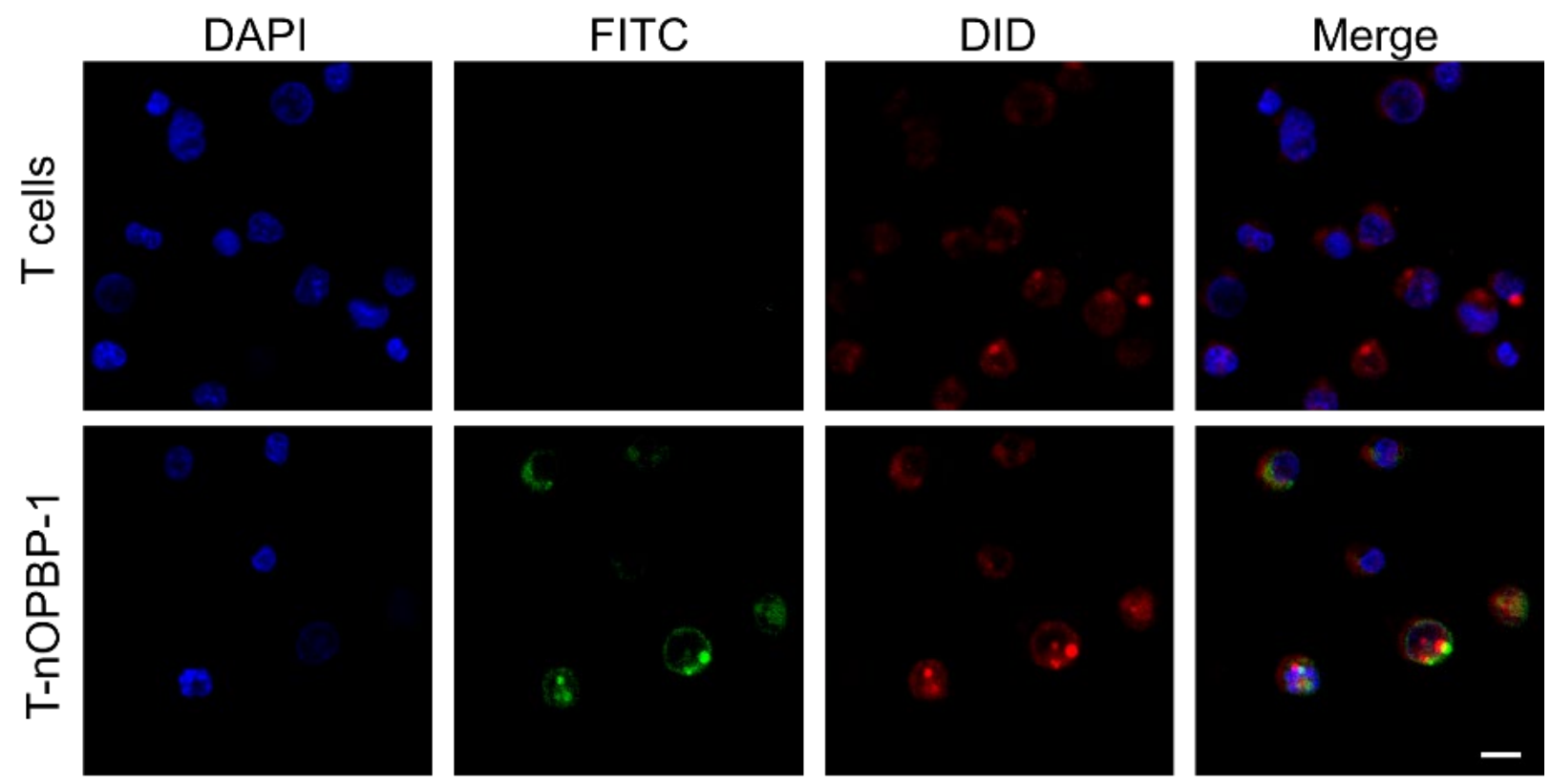

Fig. S8. Confocal imaging of T-nOPBP-1 conjugates. Unlabeled (up) and azide-labeled T cells (down) were incubated with FITC-encapsulated nOPBP-1 micelles at 37°C for 1 h. Nuclei (blue), membranes (red), and micelles (green). Scale bar, 2 μm.

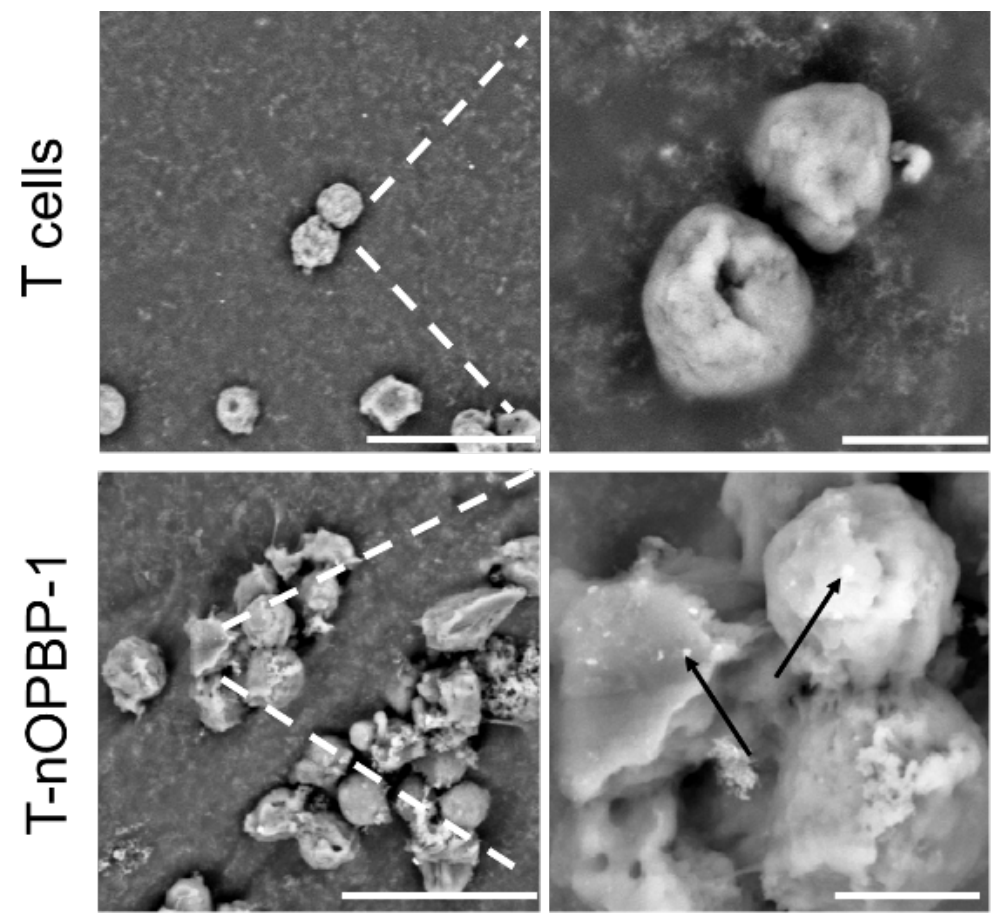

Fig. S9. SEM images of T-LEN-nOPBP-1 conjugates showing micelle attachment on T cell surfaces, absent in untreated controls. The scale bar is 20 μm in the left figure, and the bar value is 5 μm in the right figure. The image is reused from Figure 1B to facilitate comparison.

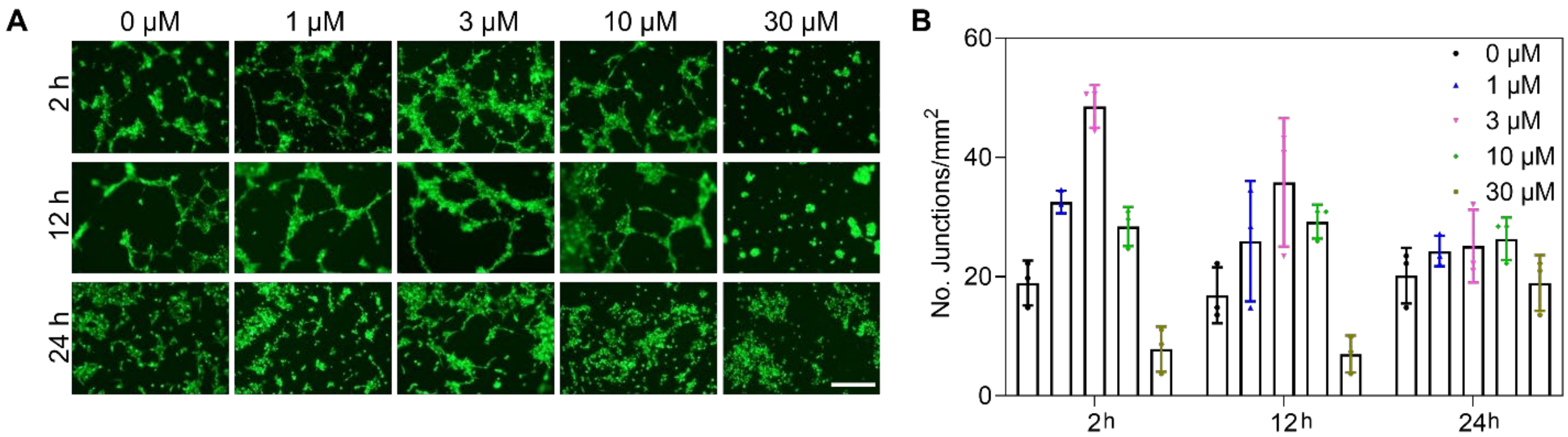

Fig. S10. LEN transiently normalizes vasculature. **(A)**, Tube formation assay of HUVECs treated with LEN (0-30 μM) for 2, 12, or 24 h. LEN promoted tube formation at 3 μM within 12 h but disrupted structures at 24 h across all doses, indicating a dose- and time-dependent effect. **(B)**, Quantification of tube nodes. Values are means ± SD; n = 3.

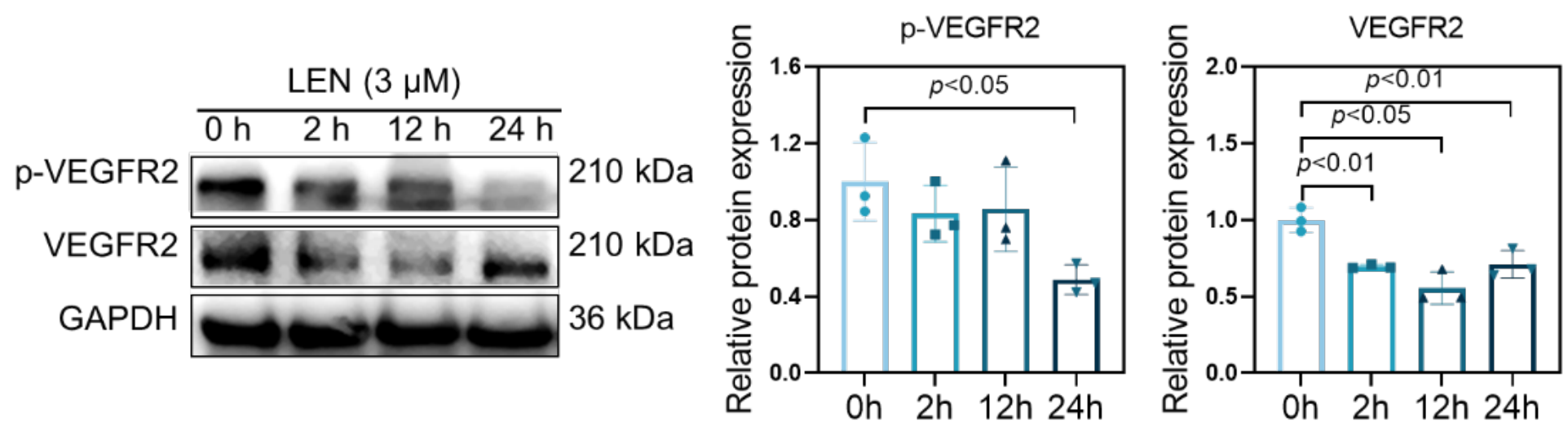

Fig. S11. Western blot analysis and quantification of total and phosphorylated VEGFR2 in endothelial cells treated with 3 μM LEN for the indicated time points, demonstrating time-dependent modulation of VEGFR2 abundance and phosphorylation status.

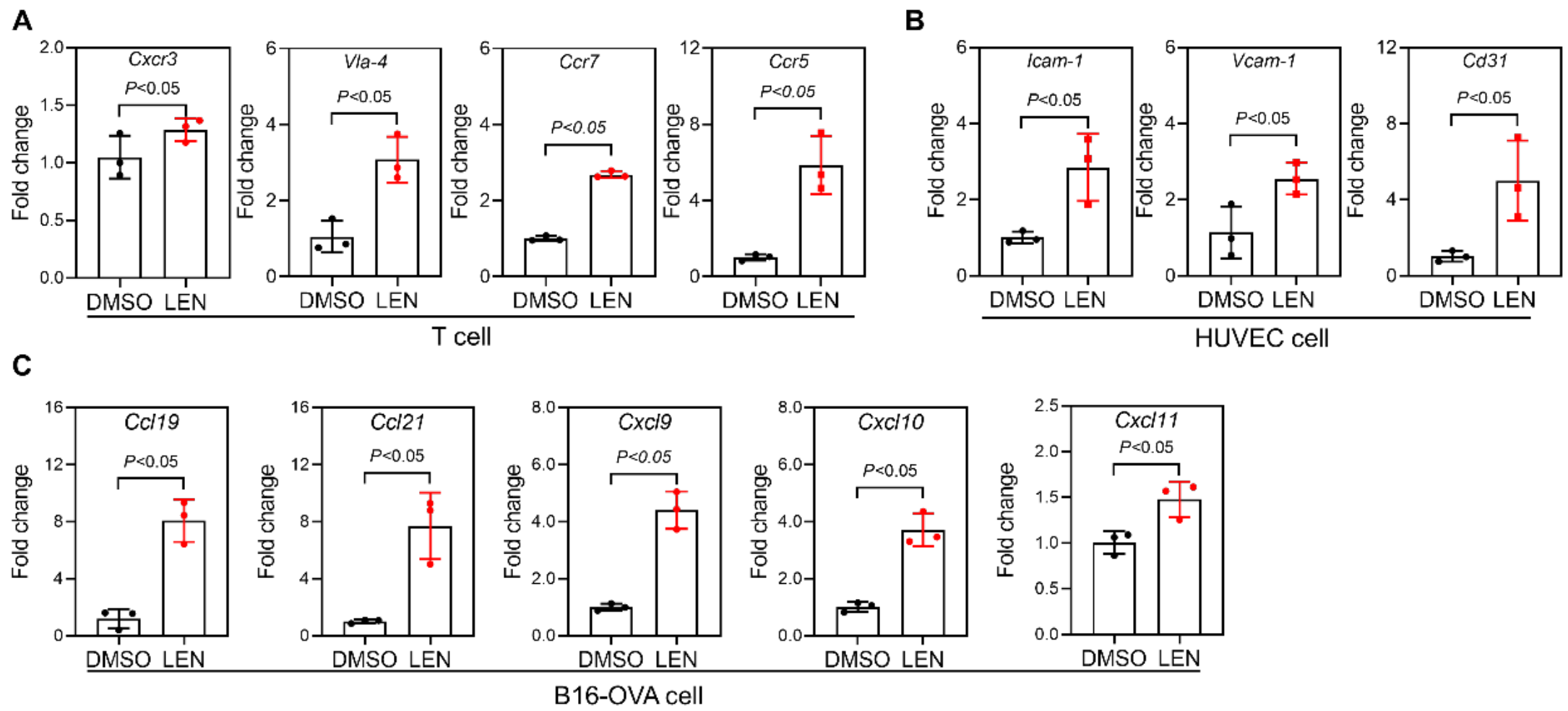

Fig. S12. qRT-PCR analysis of LEN-regulated chemotactic feedback loops. **(A)**, qRT-PCR analysis of migration-related gene expression in $CD8^+$ T after LEN treatment. **(B)**, qRT-PCR analysis of migration-related gene expression in HUVECs cells. **(C)**, chemokine-related gene expression in B16-OVA after LEN treatment. Data shown in panels (A–C) represent mean values ± SD; n = 3.

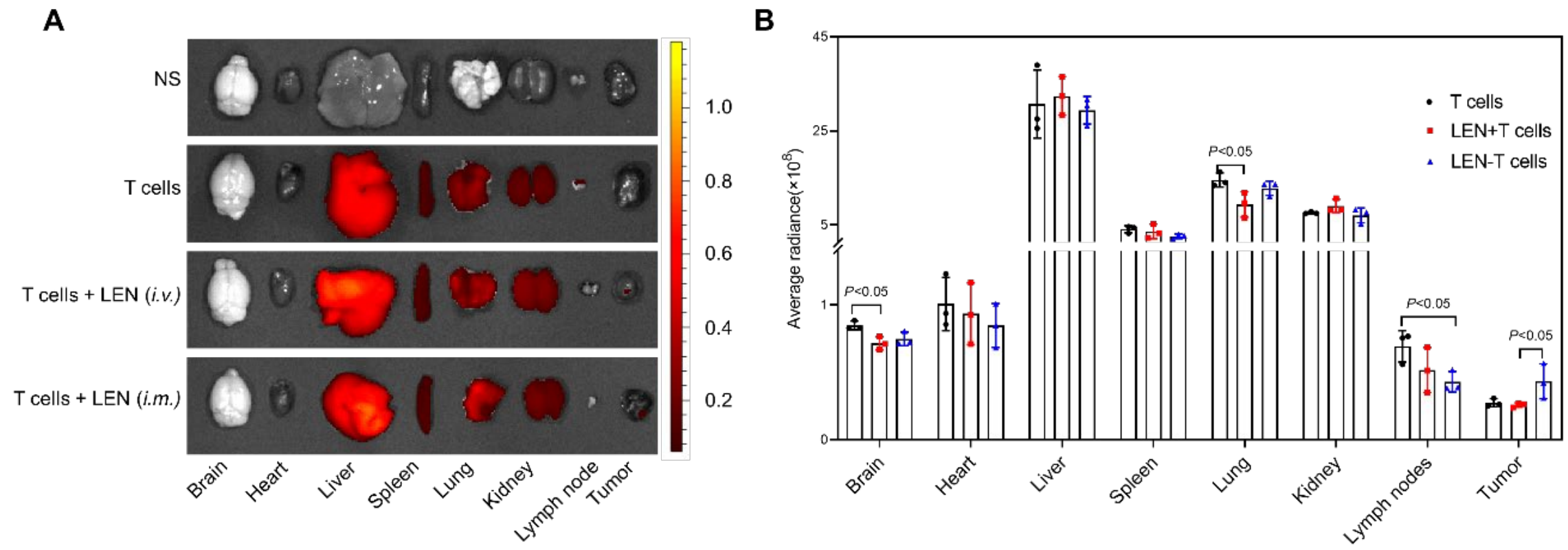

Fig. S13. LEN transiently normalizes vasculature and promotes T cell infiltration. **(A)**, Biodistribution of reinfused T cells labeled with Cy5 in B16-OVA tumor-bearing mice following intravenous or intratumoral administration of LEN. **(B)**, Quantification of Cy5-labeled T cell distribution corresponding to (A). Values are means ± SD; n = 3.

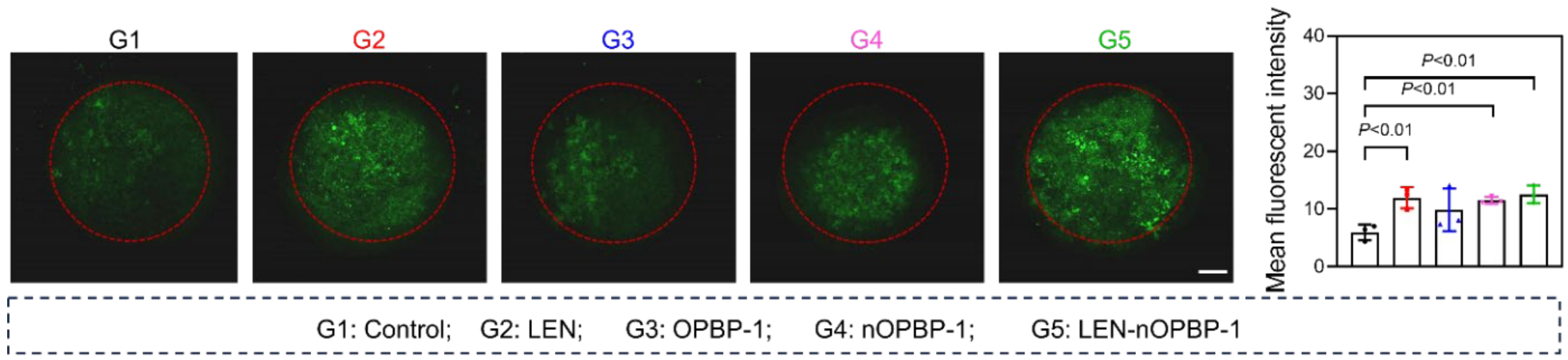

Fig. S14. Confocal imaging and quantification of CFSE-labeled T cell infiltration into 3D B16-OVA tumor spheroids. Green: T cells; red dashed line: spheroid boundary; scale bar, 200 µm. Values are means ± SD; n = 3.

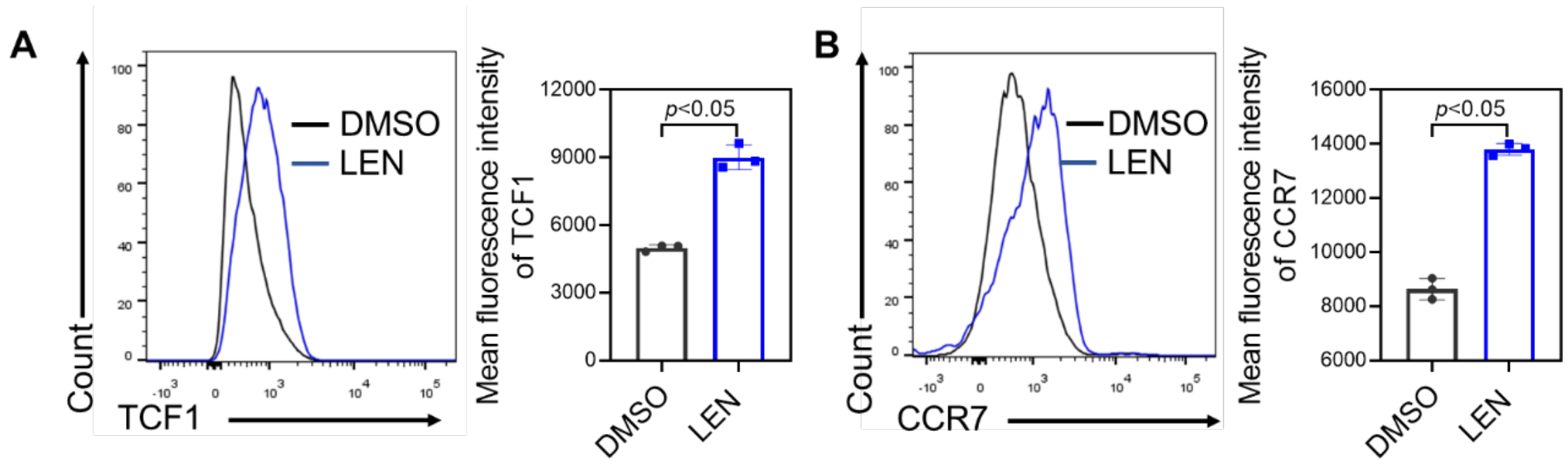

Fig. S15. Flow cytometry analysis of TCF1 **(A)** and CCR7 **(B)** expression on $CD8^+$ T cells. $CD8^+$ T cells were isolated from mouse spleens, prepared as single-cell suspensions, and pre-activated with anti-CD3 and anti-CD28 antibodies for 48 h. Activated T cells were then cultured in the presence or absence of LEN for an additional 5 days, followed by flow cytometric analysis of TCF1 and CCR7 expression. Quantification was performed based on mean fluorescence intensity. Values are means ± SD; n = 3.

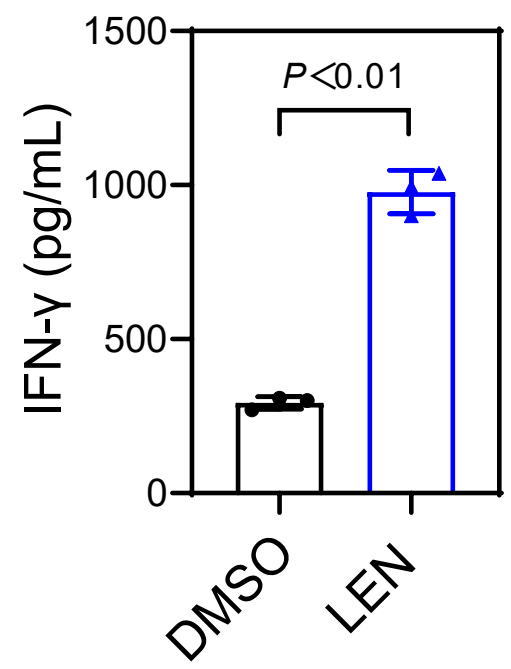

Fig. S16. IFN-γ secretion was measured by ELISA in culture supernatants from $CD8^+$ T cells pre-activated with anti-CD3 and anti-CD28 antibodies for 48 h and then treated with LEN for 3 days. Values are means ± SD; n = 3.

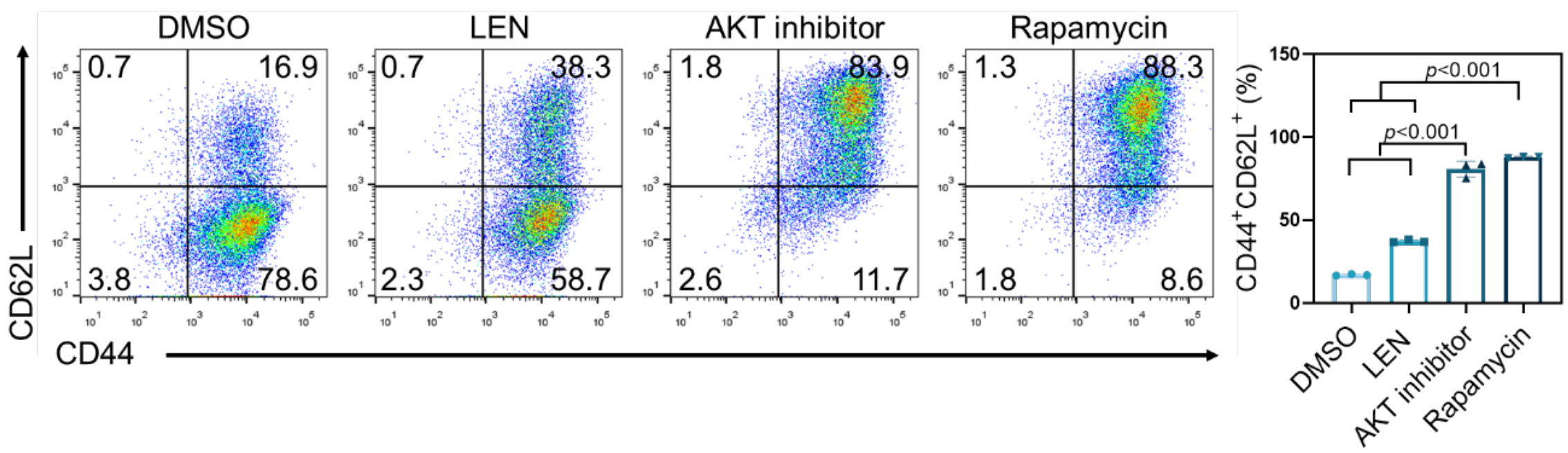

Fig. S17. Flow cytometry analysis of CD44 and CD62L expression on $CD8^+$ T cells treated with LEN (3 μM), an Akt inhibitor (HY-10249A, 1 μM), or an mTOR inhibitor (rapamycin, 10 nM) for 72 h, showing enhanced differentiation toward TCM. Values are means ± SD; n = 3.

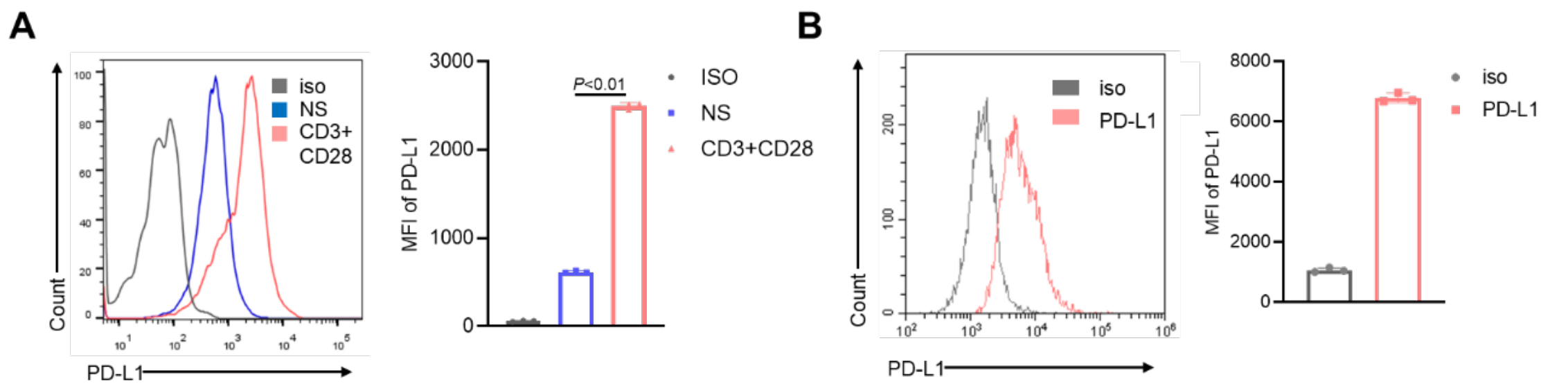

Fig. S18. (A), PD-L1 expression on activated $CD8^+$ T cells in vitro. $CD8^+$ T cells were activated in vitro with anti-CD3 and anti-CD28 antibodies. Following activation, cells were stained with antibodies against CD8 and PD-L1, and surface PD-L1 expression on $CD8^+$ T cells was analyzed by flow cytometry. **(B),** PD-L1 expression on tumor-infiltrating $CD8^+$ T cells in the MC38-OVA model. Tumors from MC38-OVA–bearing mice were excised, dissociated into single-cell suspensions, and stained with antibodies against CD3, CD8, and PD-L1. Surface expression of PD-L1 on $CD8^+$ T cells was analyzed by flow cytometry. Values are means ± SD; n = 3.

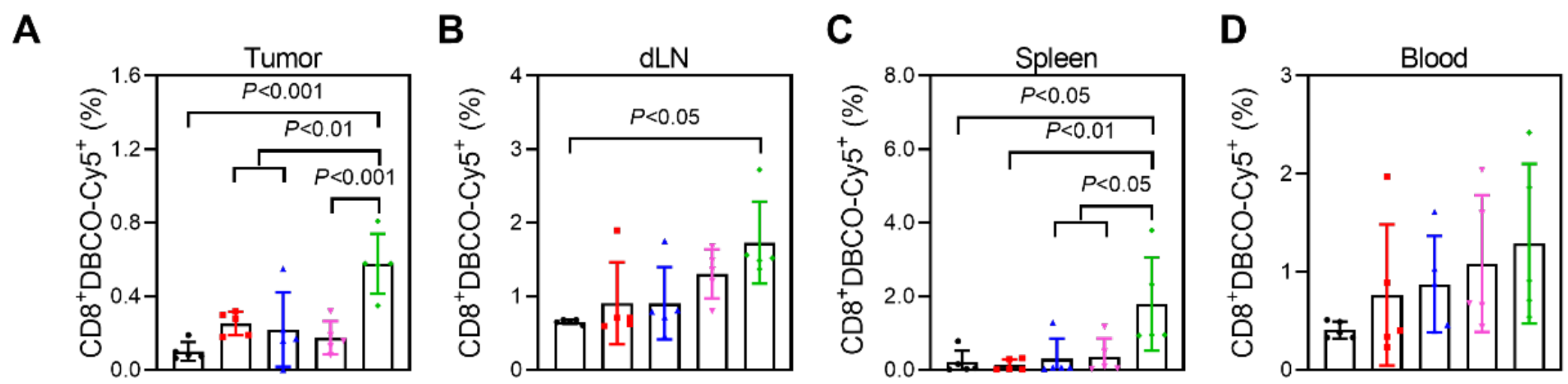

Fig. S19. Enhanced biodistribution and persistence of exogenous T cells following T-LEN-nOPBP-1 conjugation. Flow cytometric analysis of adoptively transferred Cy5-labeled OT-1 $CD8^+$ T cells among total $CD8^+$ T cells in various tissues of B16-OVA tumor-bearing C57BL/6 mice, including tumors **(A)**, draining lymph nodes **(B)**, spleens **(C)**, and peripheral blood **(D)**. Treatment groups: saline (G1, black), OT-1 T cells (G2, red), LEN-pretreated T cells (G3, blue), free LEN-nOPBP-1 micelles plus T cells (G4, purple), and T-LEN-nOPBP-1 conjugates (G5, green). Data are presented as mean ± SD; n = 5.

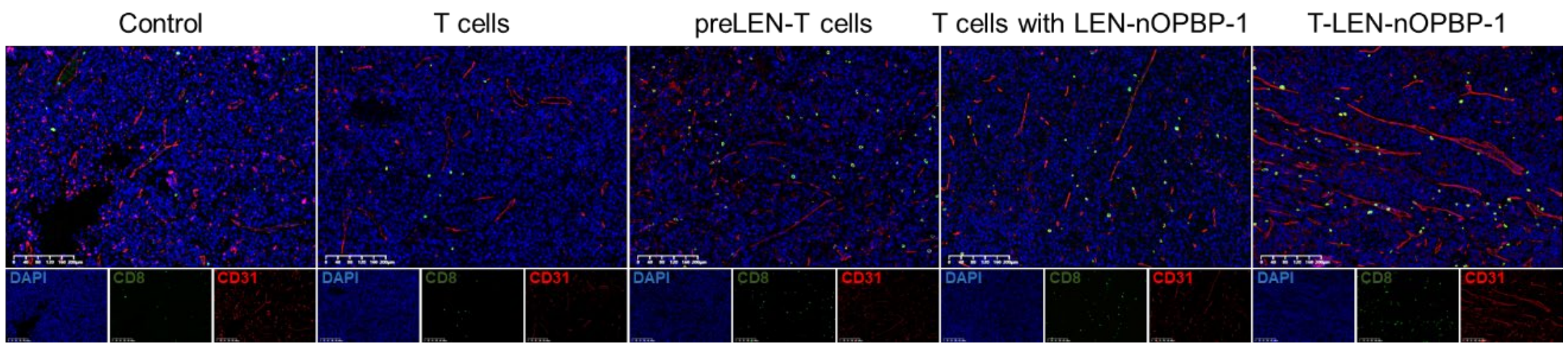

Fig. S20. Tumor sections were stained for nuclei (DAPI, blue), $CD8^+$ T cells (green), and endothelial cells (CD31, red). Scale bars, 200 µm. Tumors treated with the T cell–nanodrug conjugate showed increased intratumoral $CD8^+$ T cell density and more continuous $CD31^+$ vascular structures compared with control groups.

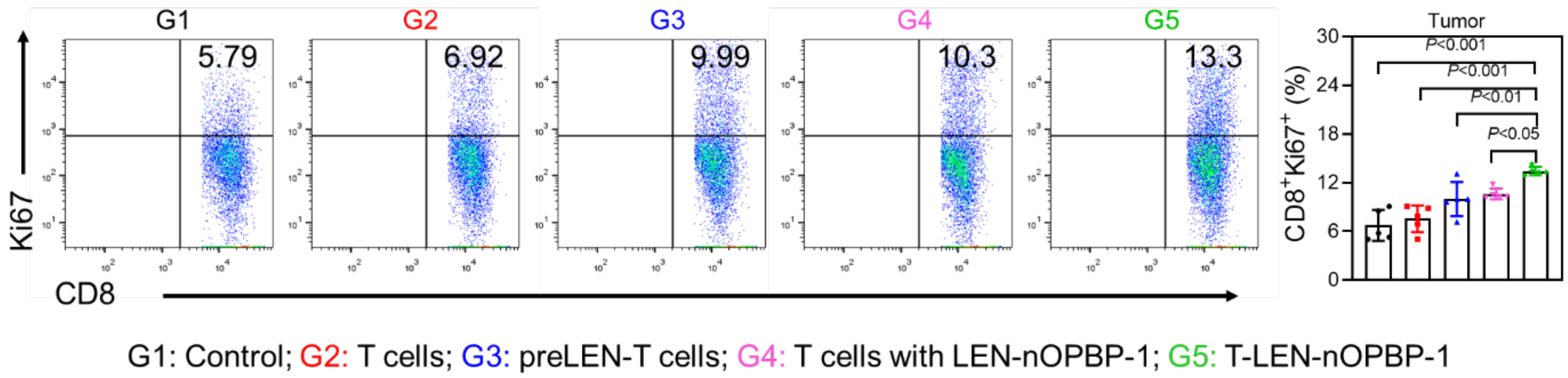

Fig. S21. T-LEN-nOPBP-1 enhances T cell function *in vivo*. Flow cytometry analysis of $CD8^+$ $Ki67^+$ T cells isolated from tumors following stimulation. Data are presented as mean ± SD; n = 5.

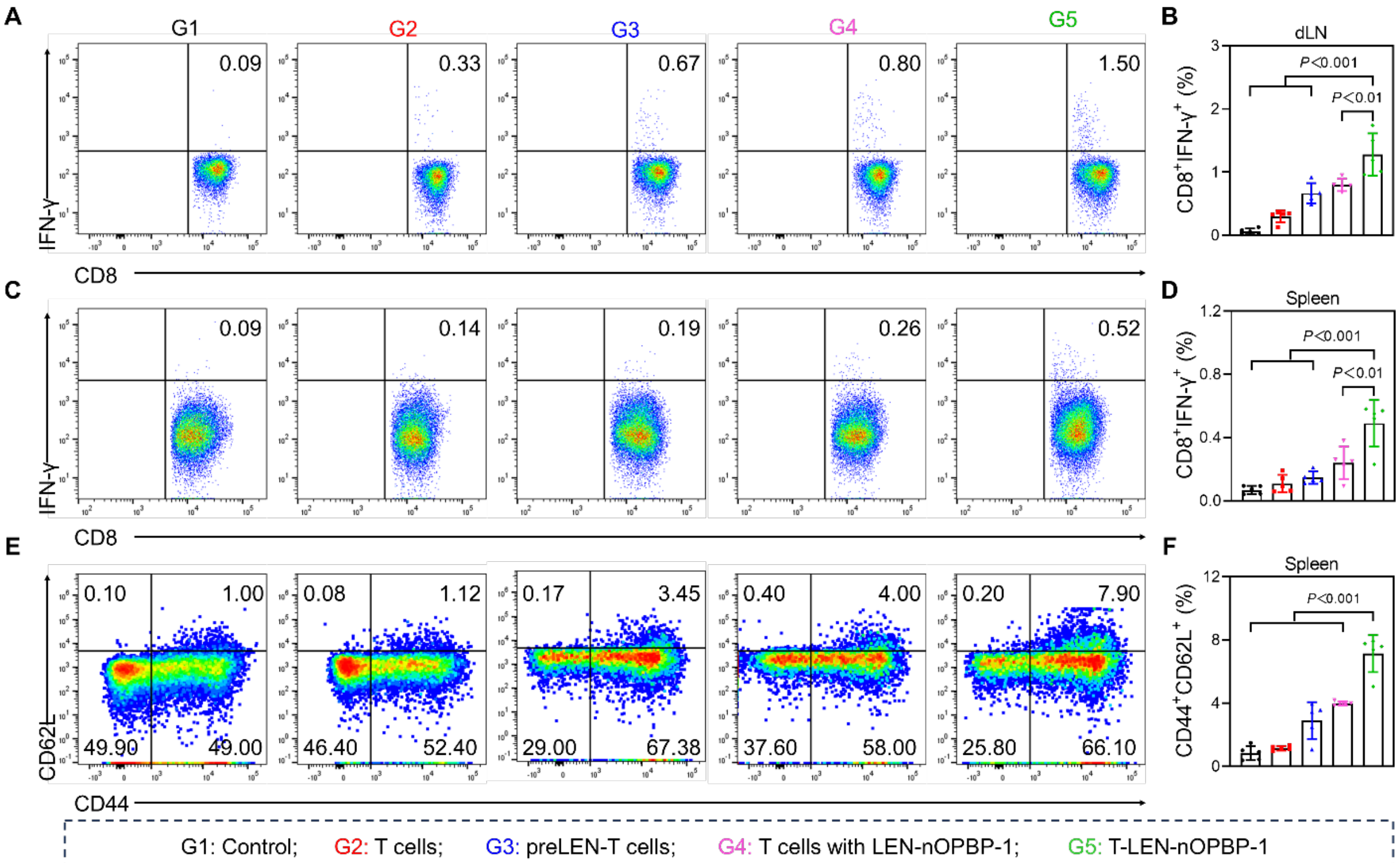

Fig. S22. T-LEN-nOPBP-1 enhances T cell function *in vivo*. Flow cytometry analysis of CD8$^+$ IFN-γ$^+$ T cells isolated from draining lymph nodes **(A)**, with corresponding quantification **(B)**, followed by analysis of cells from spleens **(C)** and their quantification **(D)** after stimulation. **(E)**, Representative flow cytometry of CD44 and CD62L expression in splenic CD8$^+$ T cells. **(F)**, Quantification of TCM (CD44$^+$CD62L$^+$) proportions from (l). Data are presented as mean ± SD; n = 5.

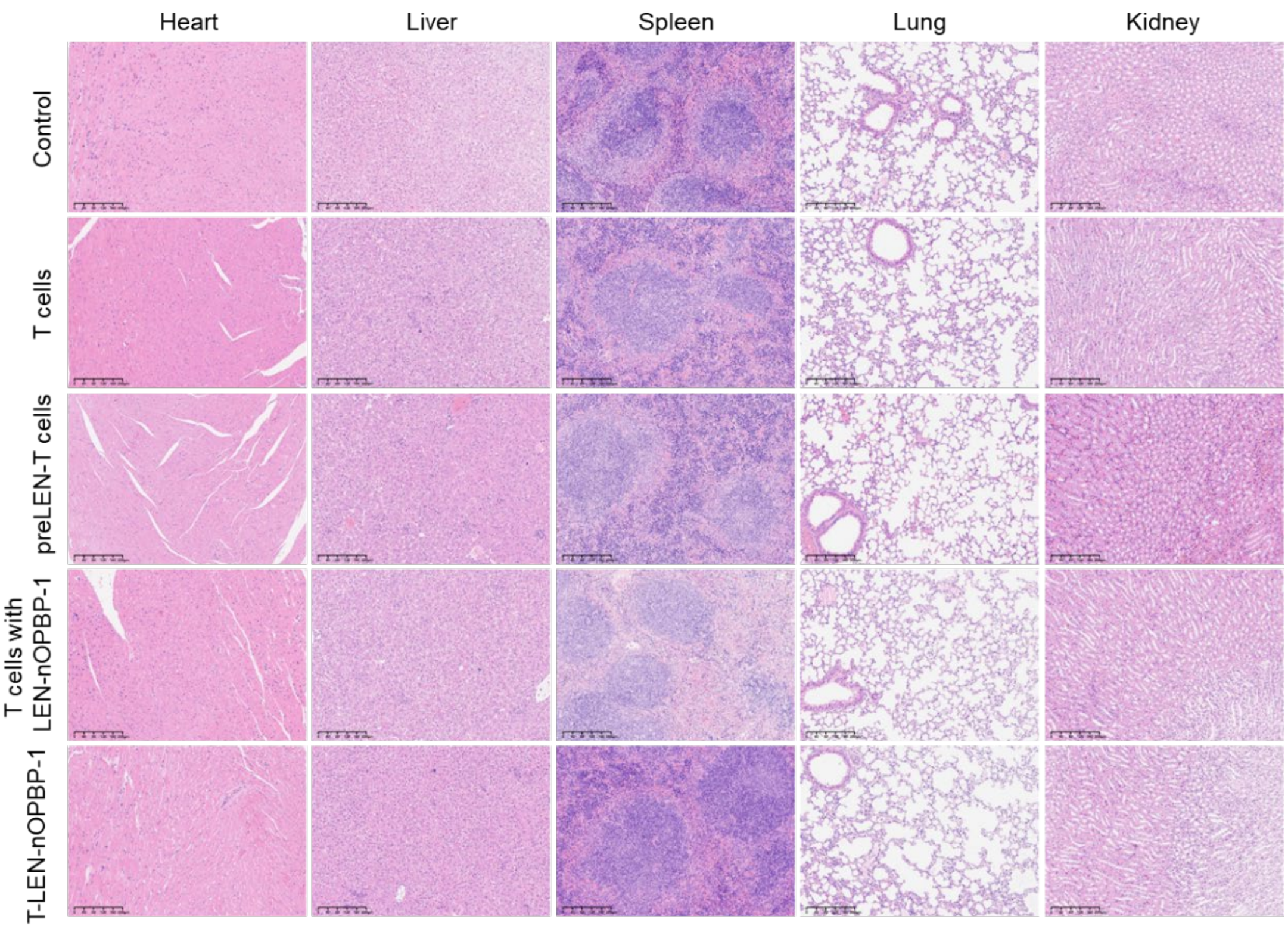

Fig. S23. The H&E staining of the organs after the treatment. Representative H&E staining of organ sections from tumor-bearing mice after the treatment. There was no obvious toxic damage in heart, liver, spleen, lung, and kidney. Scale bar: 200 μm.

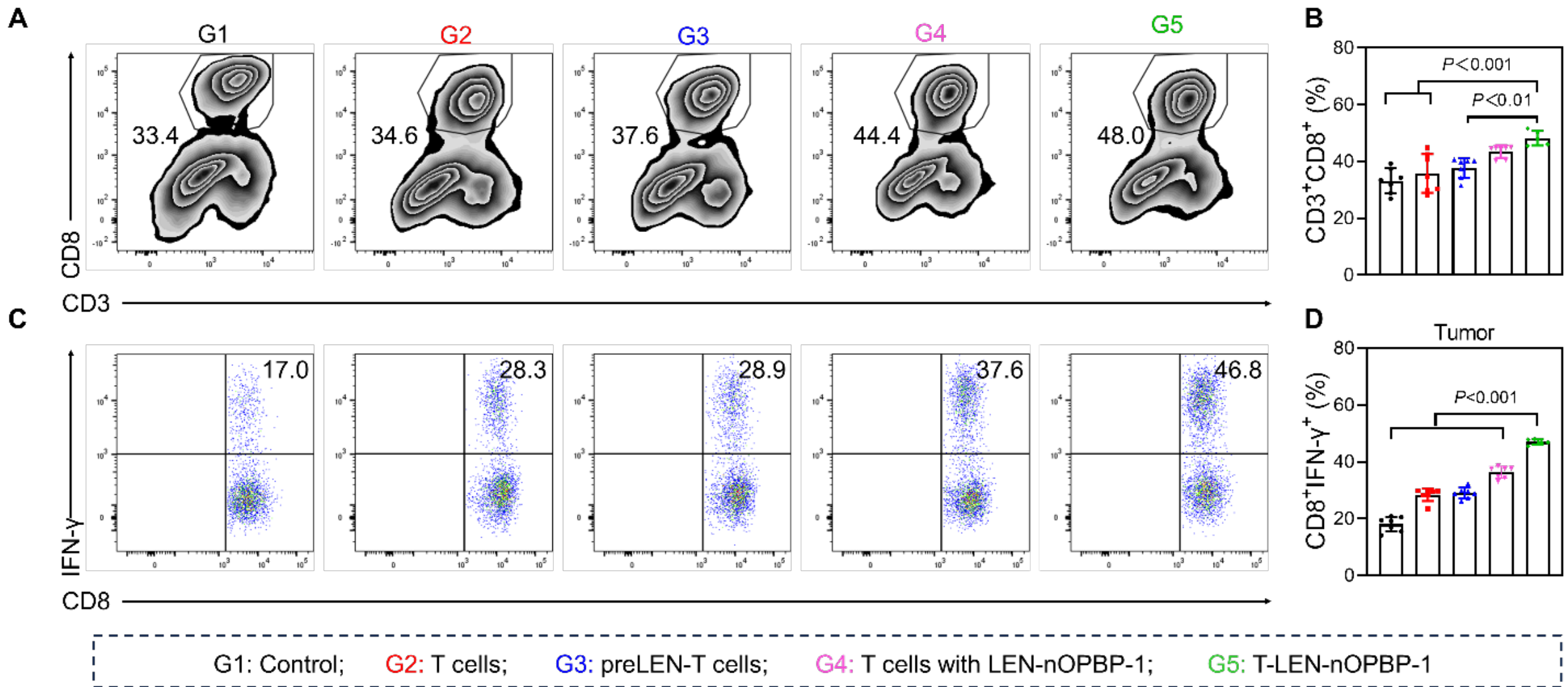

Fig. S24. Therapeutic efficacy of T-LEN-nOPBP-1 in colorectal cancer models. **(A),** Representative flow cytometry plots showing $CD3^+CD8^+$ T cells among TILs. **(B),** Quantification of intratumoral $CD3^+CD8^+$ T cell frequencies from (A). **(C),** Flow cytometry analysis of IFN-γ^+ $CD8^+$ T cells in tumors following stimulation. **(D),** Quantification of IFN-γ^+ $CD8^+$ T cells in tumors. Data are presented as mean ± SD; n = 5.

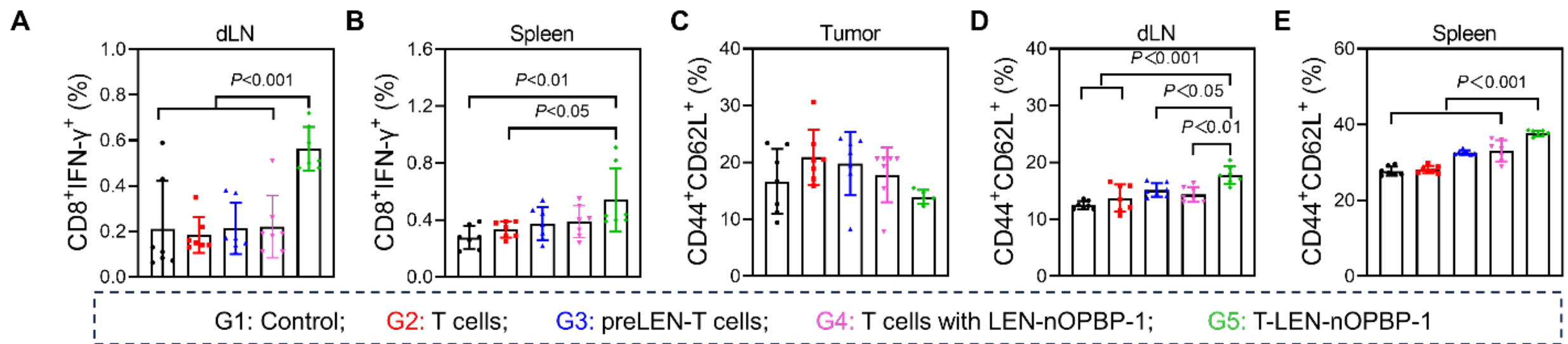

Fig. S25. Enhanced biodistribution and persistence of exogenous T cells following T-LEN-nOPBP-1 conjugation in MC38-OVA model. Flow cytometry analysis of IFN-γ^{+} $CD8^{+}$ T cells in draining lymph nodes **(A)** and Spleen **(B)**. Quantification of TCM ($CD44^{+}CD62L^{+}$) proportions in tumor **(C)**, draining lymph nodes **(D)** and spleens **(E)**. Treatment groups: saline (G1, black), OT-1 T cells (G2, red), LEN-pretreated T cells (G3, blue), free LEN-nOPBP-1 micelles plus T cells (G4, purple), and T-LEN-nOPBP-1 conjugates (G5, green). Data are presented as mean ± SD. n = 7.
